# Establishment, Characterization, and Cryopreservation of Feather Follicle Fibroblast Lines From Hyacinth Macaw (*Anodorhynchus hyacinthinus*)

**DOI:** 10.1002/cbin.70089

**Published:** 2025-10-02

**Authors:** Iara Pastor Martins Nogueira, Rachel Castro Teixeira‐Santos, Gustavo Caldeira Cotta, Wanderson Valente, John Lennon Paiva de Coimbra, Heloísa Athaydes Seabra Ferreira, Pedro Pires Goulart Guimarães, Anderson Kenedy Santos, Fernanda Mussi Fontoura, Kefany Rodrigues de Andrade Ramalho, Neiva Maria Robaldo Guedes, Samyra Maria dos Santos Nassif Lacerda

**Affiliations:** ^1^ Laboratory of Cellular Biology, Department of Morphology Institute of Biological Sciences, Federal University of Minas Gerais Belo Horizonte Minas Gerais Brazil; ^2^ Systems and Nanostructured Biophysics Laboratory, Department of Physiology and Biophysics Institute of Biological Sciences, Federal University of Minas Gerais Belo Horizonte Minas Gerais Brazil; ^3^ Genetics Department Yale School of Medicine, Yale University New Haven Connecticut USA; ^4^ Arara Azul Institute Campo Grande Mato Grosso do Sul Brazil

**Keywords:** *Anodorhynchus Hyacinthinus*, cell culture, cryopreservation, feather follicle fibroblasts (FFFs), Macaw

## Abstract

Cryopreservation, biobanking, and in vitro propagation of cells from endangered species represent key strategies for advancing biodiversity conservation. The Hyacinth Macaw (*Anodorhynchus hyacinthinus*), a flagship avian species of the Brazilian Pantanal, is among the critically vulnerable taxa that stand to benefit significantly from such cutting‐edge biotechnological interventions. This study developed and validated minimally invasive methods for isolating, culturing, and cryopreserving fibroblasts derived from feather follicles (FFFs) of Hyacinth Macaws. Cells isolated from nestlings under 100 days of age demonstrated superior yields and viability compared to older birds. Two cryopreservation media were tested—Cryomedium 1 (55% DMEM‐F12, 35% FBS, 10% DMSO) and Cryomedium 2 (90% FBS, 10% DMSO)—with Cryomedium 1 proving more effective in maintaining FFFs viability immediately post‐thaw. Several culture conditions were evaluated, including conventional plating, drop plating, gelatin coating, and supplementation with RevitaCell and bFGF. The optimal method involved conventional plating on gelatin‐coated plates with RevitaCell supplementation during the first 24 h post‐thaw. Additionally, two different culture media were tested, with KAV‐1 emerging as the best option for the long‐term propagation of Hyacinth Macaw fibroblasts. After some passages, FFFs maintained a stable karyotype of 2n = 70, expressed classical fibroblast markers such as *Vim*, *Fap*, *Acta2*, *Col1a1*, *Col1a2*, synthesized vimentin and Type I collagen in the cytoplasm, and were confirmed to be free of mycoplasma contamination. We successfully established the first primary fibroblast cell lines derived from the Hyacinth Macaw and demonstrated their efficient responsiveness to lipid nanoparticle‐mediated transfection and exogenous gene expression, representing a significant advancement toward the development of somatic reprogramming strategies. These experiments enabled the optimization of protocols for cell collection, cryopreservation, in vitro propagation, and inducing sustained heterologous expression, thereby laying a valuable foundation for the future generation of induced pluripotent stem cells (iPSCs) to support ex situ conservation efforts for this endangered species.

**Abbreviations**
Acta2: alpha smooth muscle actin (gene symbol)
ANOVA: analysis of variance
bFGF: basic fibroblast growth factor
BSA: bovine serum albumin
cDNA: complementary DNA
CIDOCD: cryopreservation‐induced delayed‐onset cell death
Col1a1: collagen Type I Alpha 1 chain (gene symbol)
Col1a2: collagen Type I Alpha 2 chain (gene symbol)
CO₂: carbon dioxide
Ct: cycle threshold
DAPI: 4′,6‐diamidino‐2‐phenylindole dihydrochloride
DLS: dynamic light scattering
DMEM‐F12: Dulbecco's modified Eagle medium/nutrient mixture F‐12
DMSO: dimethyl sulfoxide
DOPE: 1,2‐dioleoyl‐sn‐glycero‐3‐phosphoethanolamine
Fap: fibroblast activation protein (gene symbol)
FBS: fetal bovine serum
FFFs: feather follicle fibroblasts
FITC: fluorescein isothiocyanate
Gapdh: glyceraldehyde‐3‐phosphate dehydrogenase (gene symbol)
GFP: green fluorescent protein
H2DCFDA: 2′,7′‐Dichlorodihydrofluorescein diacetate
HBSS: Hank's balanced salt solution
iPSCs: induced pluripotent stem cells
IUCN: International Union for Conservation of Nature
KAV‐1: Kuwana's modified Avian culture medium‐1
LNP: lipid nanoparticles
mAb: monoclonal antibody
MEM‐Alpha: minimum essential medium alpha modification
MWCO: molecular weight cut‐off
O₂⁻: superoxide anion
PBS: phosphate‐buffered saline
PDI: polydispersity index
pDNA: plasmid DNA
PEG: polyethylene glycol
PFA: paraformaldehyde
PGC: primordial germ cell
qPCR: quantitative polymerase chain reaction
RNA: ribonucleic acid
ROS: reactive oxygen species
RPM: revolutions per minute
RT‐PCR: reverse transcription polymerase chain reaction
SA‐β‐Gal: senescence‐associated β‐galactosidase
SEM: standard error of the mean
SISBIO: Sistema de Autorização e Informação em Biodiversidade
TAE: tris‐acetate‐EDTA buffer
Tbp: TATA‐box binding protein (gene symbol)
UFMG: Federal University of Minas Gerais
Vim: vimentin (gene symbol)
w/v: weight per volume
ΔCt: delta Ct
µm: micrometer

## 1 Introduction

The situation for endangered avian species has been deteriorating globally, with Brazil notably housing one of the highest numbers of threatened species (lUCN 2021). Among these, the Hyacinth Macaw, *Anodorhynchus hyacinthinus*, the largest psittacid in the world, found mainly in central and eastern South America, faces particular challenges (Devenish et al. 2021). This species is ecologically significant due to its role in seed dispersal, as it primarily consumes two species of palm nuts, which is crucial for maintaining ecosystem dynamics (Allgayer et al. 2009). With an estimated population of around 6500 individuals, its genetic variation and population structure underscore its endangered status and highlight the urgent need for effective conservation measures (Faria et al. 2008). While various in situ conservation strategies, such as population monitoring and the installation of artificial nests, are employed—exemplified by efforts at the Arara Azul Institute in the Pantanal—these measures may not be sufficient in the face of so many negative pressures, such as wildlife trafficking, emerging diseases, habitat loss due to deforestation, and anthropological disasters, such as the significant fires that affected the Pantanal in 2019 and 2020 (dos Santos Ferreira et al. 2023a; Presti et al. 2015). Consequently, it is crucial to explore new strategies, including biotechnological advancements, that could accelerate the reproduction of these species and facilitate the effective recovery of at‐risk populations (Nogueira et al. 2024).

The establishment and thorough characterization of cell lines are fundamental prerequisites for advancing research in cellular and molecular biology within the context of biodiversity, particularly for studies on adaptive genomics, gene expression profiling, and phenotypic analyses. These cell lines also enable the development of next‐generation biological and genetic tools, including vaccine production, toxicological screening platforms, and the generation of pluripotent stem cells. Notably, the derivation of fibroblast lines from somatic tissues has become a widely adopted strategy across diverse taxa, especially in wildlife species threatened with extinction (Tavares et al. 2024; Mooney et al. 2023; Kroglund et al. 2022; Ryder and Onuma 2018). The advent of induced pluripotent stem cell (iPSC) technology in 2006 represents a transformative breakthrough, opening new frontiers for in vitro modeling, regenerative applications, and species conservation. This method allows for the genetic reprogramming of somatic cells into pluripotent cells, which have the capacity for self‐renewal, genetic stability, and the ability to differentiate into any cell type from all three germ layers, both in vitro and in vivo (X. Liu et al. 2008; Yamanaka 2008). In the context of animal reproductive science, iPSCs offer a significant opportunity for the conservation of rare populations (Hayashi et al. 2022; Stanton et al. 2019; Zywitza et al. 2022). They enable the preservation of genetic material from threatened species without the need to sacrifice individuals, by allowing the reprogramming of adult somatic cells obtained from non‐invasive tissue collections (Dicks et al. 2021; Stanton et al. 2019). In avian species, the generation of iPSCs has been demonstrated from embryonic fibroblasts of chicken (*Gallus domesticus*) and quail (*Coturnix coturnix*) (Katayama et al. 2018; Lu et al. 2012, 2015; Rosselló et al. 2013), as well as from fibroblasts derived from feather follicle cells in both domestic and wild birds (Katayama et al. 2022; Kim et al. 2017). In future applications, the reprogramming of avian somatic cells into iPSCs, followed by their directed differentiation into primordial germ cells (PGCs), may represent a powerful strategy to enable xenotransplantation approaches—such as the generation of germline chimeras (Choi et al. 2015; T. S. Park et al. 2003; Woodcock et al. 2019)—for use in reproductive technologies. This innovative perspective is likely to be increasingly explored by research groups aiming to expand the frontiers of conservation and assisted reproduction.

This study aimed to establish and optimize protocols for the collection, isolation, cryopreservation, and in vitro cultivation of fibroblast cell lines derived from feather follicles of Hyacinth Macaws, demonstrating also their capacity to express exogenous genes via nanocarrier‐mediated transfection. As biotechnological tools for avian cellular manipulation advance, the establishment of standardized biobanks becomes essential to ensure the long‐term preservation and accessibility of genetic resources from threatened populations. The Hyacinth Macaw, recognized as a flagship species with strong and research funding appeal, also functions as an umbrella species for the conservation of other endangered avian taxa (Guedes et al. 2022; Vilaça et al. 2024). Accordingly, the methodologies and technological advances achieved using this model may serve as a platform to support broader conservation initiatives targeting phylogenetically or ecologically related bird species.

## 2 Materials and Methods

### 2.1 Sample Collection

Feather follicle cells are generally acquired from the calamus of actively growing feathers collected from the primary and secondary remiges of the avians wing (Cardoso et al. 2020; XI et al. 2003). In this study, due to the single access opportunity to wild specimens, we opted to collect samples from nestling Hyacinth Macaws that were still in the feathering stage, ensuring the feathers were actively growing. A partnership with the Arara Azul Institute was established and two collection events took place in January over consecutive years (2022–2023) at the Caimã Biological Reserve in Pantanal, Mato Grosso do Sul, Brazil (19°57′15.4″ S 56°18′15.4″ W) (SISBIO Number: 83255‐1). In the first year, 10 animals were sampled, while in the second year, 11 animals were accessed. In addition to samples from free‐living birds, feathers were also collected from 3 Hyacinth Macaws chicks housed at the Jardim Zoológico of the Fundação de Parques e Zoobotânica de Belo Horizonte, Minas Gerais, Brazil. All experiments were approved by the Animal Use Ethics Committee of the Federal University of Minas Gerais (UFMG) under protocol 71/2022. The birds sampled, aged between 50 and 115 days, were carefully restrained and temporarily removed from their nests for the purpose of this study. Ice was applied to each wing for 2 min, followed by cleaning of the site with 70% alcohol before feather plucking. One feather from each wing was collected and the calamus was separated from the plumage, washed in PBS (Sigma‐Aldrich) with 2% antibiotic‐antimycotic solution (Gibco), and maintained in DMEM‐F12 (Gibco) with 10% fetal bovine serum (FBS) (Nova Biotecnologia) and 1% antibiotic‐antimycotic solution (10,000 units/mL of penicillin, 10,000 μg/mL of streptomycin, and 25 μg/mL of amphotericin B—Gibco). All samples were kept at 4°C during the collection and transport period.

### 2.2 Sample Processing and Age‐Related Yield Analysis

Each bird sample was processed individually. The calamus was washed three times in PBS with 2% antibiotic‐antimycotic solution in a Petri dish. Subsequently, using Castroviejo scissors, the calamus was carefully opened in the same dish, and the pulp was meticulously removed intact. Feather pulp was transferred to a new dish containing a 0.1% collagenase Type IV solution (Sigma‐Aldrich) in DMEM‐F12 basal media. The samples were minced with scalpels and dissociated by pipetting, and then incubated in a mechanical shaker at 38°C with a rotation speed of 150 RPM for 60 min. After the incubation period, further dissociation by pipetting was performed, and the digested tissue was filtered through a 40 µm cell strainer (Corning) into a centrifuge tube. The enzyme activity was halted by adding a 1:1 volume of DMEM‐F12 with 20% FBS to the collagenase solution. The feather follicle cells were then centrifuged at 300*g* for 12 min at room temperature. The supernatant was discarded, and the resulting pellet was resuspended and cautiously homogenized in 1 mL of DMEM‐F12 with 10% FBS and 1% antibiotic‐antimycotic. In second year of sample collection, following cell counting using a Neubauer chamber with Trypan Blue 0.4% (Gibco) stain, the total and viable cell numbers were correlated with the age categories of the nestlings: young chicks less than 100 days (*n* = 5) old and those older than 100 days (*n* = 6).

### 2.3 Primary Culture

During the first‐year collection feather follicle cells from each Hyacinth Macaw nestling were plated in T25 cm^2^ flasks (Sarstedt) and maintained in DMEM‐F12 with 10% FBS and 1% antibiotic‐antimycotic solution. In the second year of collection, half of feather follicle cells from each bird were plated directly into 24‐well plates (Sarstedt), while the remaining half were plated into 24‐well plates pre‐treated with 0.2% gelatin (from porcine skin, Sigma‐Aldrich) at 37°C for 2 h. Additionally, feather follicle cells from each bird were divided into two test groups—spread plating and drop plating (Martin and Rubin 1974). For this, half of the cells were distributed using the standard seeding method spreading the cell through the entire well area, while the other half were centrifuged (300*g* for 10 min) and resuspended in 20 µL of culture medium to execute the drop plating method. Specifically, a single drop was placed in the center of the well and incubated for 1 h, after which the appropriate volume of culture medium was added. Aiming at the enrichment of fibroblasts (Jozefczuk et al. 2012), after 2 h of incubation at 37°C and 5% CO₂, the supernatant containing non‐adherent cells was removed. The feather follicle‐derived fibroblasts (FFFs) were washed twice with PBS, and fresh culture medium (DMEM‐F12 supplemented with 10% FBS and 2% antibiotic‐antimycotic) was added. The medium was completely replaced every 24 h, and photos were daily taken for 7 days using Motic AE31E microscope and Moticam 2300 camera.

### 2.4 Cryopreservation and Thawing Evaluation

Following the second passage at 80% of confluency, the Hyacinth Macaw FFFs were detached with 0.25% (w/v) Trypsin (Gibco) for 90 s and neutralized in DMEM‐F12 with 20% FBS and 1% antibiotic‐antimycotic. FFFs were then centrifuged at 500*g* for 12 min at room temperature. The supernatant was discarded, and the pellet was resuspended in 1 mL of culture medium. Cell counting was performed and, approximately 5 × 10^5^ cells from each bird were frozen in 1 mL of cryomedium. The samples were divided into two groups to test different cryopreservation media containing dimethyl sulfoxide (DMSO): Cryomedium 1, composed of 55% DMEM‐F12, 35% FBS and 10% DMSO and Cryomedium 2, composed of 90% FBS and 10% DMSO (Moore et al. 2006; Jimenez and Williams 2014; Cardoso et al. 2020; Chaipipat et al. 2021; Katayama et al. 2022).

Cryotubes were placed in a Mr. Frosty (Nalgene) container and cooled in a −80°C freezer for at least 12 h before being stored in liquid nitrogen for long‐term preservation. After 15 days, the FFFs were thawed for 90 s in a water bath and washed with DMEM‐F12 with 20% FBS and 1% antibiotic‐antimycotic. The cells were then centrifuged at 350*g* for 7 min at RT and the pellet was resuspended in 1 mL of culture medium. Using cell counting with Trypan Blue in a Neubauer chamber, the total cell recovery and cell viability for each cryomedium were determined.

Following the observation of significant cell death within the first 24 h post‐thawing, the effect of RevitaCell was assessed. To address this, a new thawing experiment was conducted using FFFs previously cryopreserved in Cryomedia 1 and 2. In contrast to the previous protocol, RevitaCell Supplement 100X (Gibco) was added at a final concentration of 10 µL/mL to both KAV‐1 medium (MEM‐Alpha supplemented with 5% chicken serum, 5% FBS, 1% antibiotic‐antimycotic, 2 mM GlutaMAX, and 1% non‐essential amino acids [Gibco]) and DMEM‐F12 medium (supplemented with 10% FBS and 1% antibiotic‐antimycotic). These RevitaCell‐supplemented media were used during the cryopreservation medium wash step and subsequently maintained throughout the first 24 h of FFFs culture. After this period, the media were replaced, and cell viability was evaluated using the CellTiter‐Blue Cell Viability Assay (Promega). Fluorescence measurements were obtained using BioTek Cytation 5 Cell Imaging Multi‐Mode Reader (Agilent Technologies) to assess cell viability across the experimental groups.

### 2.5 Apoptosis Detection

To investigate whether RevitaCell modulates apoptotic pathways involved in cryopreservation‐induced cell death, the activities of Caspase‐8 and Caspase‐3 were assessed 24 h after thawing of FFFs. Caspase activity was measured using the Caspase‐Glo 8 and Caspase‐Glo 3/7 Assay Systems (Promega, Mannheim, Germany), following the manufacturer's protocol. Thawed cells (10⁴ cells/well) were cultured in 96‐well plates with 100 μL of KAV‐1 medium, either with or without RevitaCell, for 24 h in sextuplicate. Subsequently, 100 μL of the respective Caspase‐Glo reagent was added to each well. Plates were gently mixed for 2 min, incubated at RT in the dark for 1 h, and luminescence was recorded using the BioTek Cytation 5 Cell Imaging Multi‐Mode Reader (Agilent Technologies).

### 2.6 Measurement of Mitochondrial Superoxide and Reactive Oxygen Species (ROS) Production

The intracellular production of ROS was evaluated using the fluorescent dye 2′,7′‐dichloro‐dihydrofluorescein diacetate (H_2_DCFDA, Invitrogen) according to the manufacturer's indication. Post‐thaw FFFs (10^5^ cells/well) were cultured with or without RevitaCell supplementation, for 24 h in 48‐well plates in sextuplicate. Subsequently, the cells were incubated with H_2_DCFDA (10 μM) in serum‐free medium for 30 min at 37°C, protected from light. Next, the dye was removed and the cells washed three times with PBS and the fluorescence intensity was measured on the BioTek Cytation 5 Cell Imaging Multi‐Mode Reader (Agilent Technologies) at 495/527 nm. The production of intracellular superoxide anions (O_2_^−^) was investigated using the MitoSOX Red Mitochondrial Superoxide Indicator kit (ThermoFisher Scientific, USA) according to the manufacturer's instructions. After 24 h of thawing FFFs cultured with or without RevitaCell were incubated with MitoSOX Red (500 nM) prepared in HBSS buffer for 30 min at 37°C in the dark. After incubation, the cells were washed three times with HBSS and analyzed. Fluorescence was measured on the BioTek Cytation 5 Cell Imaging Multi‐Mode Reader (Agilent Technologies) at 531/593 nm.

### 2.7 Growth Curve Behavior in Different Media and Senescence Assay

To assess the eventual impact of cryopreservation media and identify the most effective media for Hyacinth Macaw cell propagation, a cell growth curve experiment (*n* = 6 birds) was conducted. Following the initial thawing experiment, 2.5 × 10^4^ FFFs were seeded in sextuplicate in 12‐well plates pre‐treated with 0.2% gelatin. The culture media were completely replaced and replenished daily, and the cells were photographed every other day using a Motic AE31E microscope with a Moticam 2300 camera.

Four experimental groups were established. The two culture media tested were DMEM‐F12 supplemented with 10% FBS and 1% antibiotic‐antimycotic, and KAV‐1 [25]. The groups were defined as follows:

‐ 1.D: FFFs cryopreserved in Cryomedium 1 and cultured in DMEM‐F12 medium.

‐ 2.D: FFFs cryopreserved in Cryomedium 2 and cultured in DMEM‐F12 medium.

‐ 1.K: FFFs cryopreserved in Cryomedium 1 and cultured in KAV‐1 medium.

‐ 2.K: FFFs cryopreserved in Cryomedium 2 and cultured in KAV‐1 medium.

On the fifth day post‐thaw, cells from each well were trypsinized, counted using a Neubauer chamber, and replated. This procedure was similarly conducted on Days 10 and 15. Following the final cell count, at the end of the 15‐day period, FFFs from each sample were replated.

The subsequent day, a cellular senescence assay was performed using the CellEvent Senescence Green Detection Kit (Invitrogen) to assess the activation of senescence‐associated beta‐galactosidase (SA‐β‐Gal). Before the beginning of the assay, the working solution was prepared by warming the CellEvent Senescence Buffer to 37°C and then diluting CellEvent Senescence Green Probe (1000 ×) on it. FFFs were plated into a 96‐well plate and incubated overnight. After washing the cells with PBS, they were fixated with 2% paraformaldehyde (PFA, Sigma) for 10 min at room temperature and protected from light. The plate was then washed with 1% BSA in PBS and, to each well, were added prewarmed working solution. The plate was covered in aluminum foil and incubated for 2 h at 37°C. After the incubation time, the working solution got removed and the wells were washed three times with PBS. To allow the fluorescent and SA‐β‐Gal activity detection, the assay was made by using an Alexa Fluor 488/FITC filter set. Images and fluorescence quantification were evaluated using BioTek Cytation 5 Cell Imaging Multi‐Mode Reader (Agilent Technologies). Further, new growth curve of 15 days was assessed for FFFs at passages #10 and #3 in KAV‐1 medium supplemented or not with 10 ng/mL of human basic fibroblast growth factor (bFGF, Sigma).

### 2.8 Immunofluorescence

Vimentin, Type I collagen (positive markers) and cytokeratin (negative marker) protein detections were used to characterize the Hyacinth Macaw FFFs. Cells cultured in DMEM‐F12 and KAV‐1 were seeded in 48 well plates and, after 24 h, fixed with 4% PFA. Following three 5‐min washes with PBS, permeabilization was performed using 0.3% Triton X‐100 (Sigma) for 10 min, followed by another washing cycle. A 1% bovine serum albumin (BSA, Sigma) solution was applied for 1 h to block non‐specific binding sites. FFFs were then incubated with a 1:100 dilution of rabbit anti‐vimentin monoclonal antibody (Invitrogen), goat anti‐collagen 1 (Santa Cruz Biotechnology), and mouse anti‐cytokeratin (Santa Cruz Biotechnology) overnight at 4°C. Non‐related antibody (rabbit anti‐neurophysin mAb—Santa Cruz Biotechnology) and omission of primary antibody were used as negative controls. The primary antibody was removed, washed 3 times in PBS and the samples were incubated with secondary antibodies: donkey anti‐rabbit Alexa Fluor 488 (Invitrogen), donkey anti‐goat Alexa Fluor 546 (Invitrogen) or goat anti‐mouse Alexa Fluor 633 (Invitrogen) for 3 h. After washing with PBS for 3 times, 4′,6‐diamidino‐2‐phenylindole dihydrochloride (DAPI, Sigma) was applied for 5 min for nuclear staining, and the samples were maintained in PBS. Images were captured using BioTek Cytation 5 Cell Imaging Multi‐Mode Reader (Agilent Technologies), utilizing BioTek Gen‐5 (Agilent Technologies) capture software.

### 2.9 Primers Design and qPCR for Fibroblast Markers

Five genes were chosen to conduct our qPCR experiments, based on the current applied markers for fibroblast characterization (J. Chen et al. 2023). Primers were designed based on the alignment of known sequences retrieved from the NCBI database for the following genes: *Col1a1*, *Col1a2*, *Fap*, *Vim*, *Acta2*, and *Tbp* (Table 1). The alignment included sequences from members of the Psittacidae family (*Melopsittacus undulatus, Nestor notabilis, Neopsephotus bourkii, Pezoporus wallicus, Pezoporus occidentalis, Pezoporus flaviventris, Strigops habroptila, Lathamus discolor*), followed by the analysis of conserved regions within the family.

**Table 1:** Primers for qPCR.

Target	Forward primer (5′ – 3′)	Reverse primer (5′ – 3′)	Category
*Tbp*	CAGCAAGCAACACAGGGAAC	TGGAGAGGGGTACAGAGGTG	Housekeeper gene
*Acta2*	TGTGTTATGTGGCCCTGGAC	GTGATCACCTGGCCATCAGG	Fibroblast marker
*Vim*	GGAGGAAGCTGAGAACACCC	AAGGTCAAGACGTGCCAGAG	Fibroblast marker
*Col1a1*	AACGCTGAGATCCCCTTTGG	CAGCGCTTTCTGGGTAGACA	Fibroblast marker
*Col1a2*	GGGCCTAAAGGACCTAAGGG	GCACCAGGGAAACCAGTCAT	Fibroblast marker
*Fap*	TGGGATTCCTGACTGGGTCT	CCACTTGGAGACCACCACAA	Fibroblast marker

FFFs lines from six birds were cultured in KAV‐1 medium until reaching confluence. Subsequently, they were trypsinized, and the pellet was collected by centrifugation at 500*g*. The samples were immediately resuspended in 500 µL of RNA Later (Sigma) and stored at −20°C until processing. RNA quantification was performed using a Nanodrop 2000 spectrophotometer (ThermoFisher), confirming adequate RNA concentration and optimal purity levels based on the 260/280 nm absorbance ratio. For cDNA synthesis, 200 ng of RNA per sample was reverse‐transcribed using the SuperScript III First‐Strand Synthesis System for RT‐PCR (Invitrogen). The resulting cDNA samples were stored at −20°C until qPCR analysis. Each qPCR reaction was performed using 5 ng of cDNA and 500 nM of forward and reverse primers in a reaction mixture prepared with GoTaq qPCR Master Mix (Promega). All reactions were conducted in triplicates. The qPCR reactions were performed on a QIAquant 96 5 Plex thermal cycler (Qiagen), with fluorescence captured during the extension and melting phases. The qPCR cycling conditions included an initial polymerase activation at 95°C for 2 min, followed by 40 cycles of 15 s at 95°C and 1 min at 60°C, and a subsequent melting curve analysis from 72°C to 95°C. For each target gene, ΔCt values were calculated by normalizing the Ct values to the housekeeping gene *Tbp*. The average expression levels of target genes for each independent FFF line were determined using the 2^−ΔCt^ method and expressed on a log_2_ scale, as recommended by Schmittgen and Livak (2008).

### 2.10 Karyotyping of FFF Cell Lines

FFFs were cultured in T25 cm² flasks containing KAV‐1 medium for 72 h before mitotic arrest. Chromosome preparations were performed according to established protocols (Güney‐Esken et al. 2021; Moralli et al. 2011), with appropriate modifications. On the day of karyotype analysis, cultures were washed with PBS, and the medium was replaced. Subsequently, 10 µL of KaryoMAX Colcemid Solution (Gibco) were added to each flask, and the cells were incubated at 37°C in a humidified atmosphere with 5% CO₂ for 30 min to arrest cells in metaphase. Following incubation, the culture medium containing Colcemid was collected into a 15 mL conical centrifuge tube. Adherent cells were trypsinized for 3 min, the enzymatic reaction was neutralized with complete KAV‐1 medium, and the resulting cell suspension was transferred to the same tube containing the previously collected medium. The cells were centrifuged at 300*g* for 7 min, and the supernatant was carefully discarded. The cell pellet was gently resuspended in 10 mL of pre‐warmed (37°C) 0.075 M potassium chloride (KCl) hypotonic solution and incubated at 37°C in 5% CO₂ for 18 min to induce cellular swelling. After hypotonic treatment, 100 µL of Carnoy's fixative (methanol:glacial acetic acid, 3:1) was added dropwise to the suspension. The tube was gently inverted once and allowed to stand for 1 h at room temperature to complete fixation. FFFs were then centrifuged at 500*g* for 8 min. The supernatant was removed, and 1 mL of Carnoy's fixative was added, resuspending the pellet. The suspension was centrifuged at 300*g* for 5 min, the supernatant was removed, and the pellet was resuspended again in 1 mL of Carnoy's fixative. This process was repeated three times. After the washes, the pellet was resuspended once more in 1 mL of fixative. A total of 20 µL of the chromosome preparation was dropped onto pre‐cleaned glass slides from a height of about 10 cm. After the material had completely dried, the slides were stained with 5% Giemsa for 5 min, mounted with glass coverslips using Entellan (Merck), and analyzed on a light microscope at ×100 objective (Olympus BX43). Seventy metaphases were counted. One metaphase was selected for karyotype alignment and documentation in the results, using Adobe Photoshop 2021 software.

### 2.11 Mycoplasma Detection

The culture medium was collected from FFFs at passages #2, #4, and #8 for the KAV‐1 medium, and at passage #8 for the DMEM‐F12 medium. These samples were stored at −20°C until testing. Due to its superior sensitivity, simplicity, and rapid turnaround time, we employed a PCR‐based assay for mycoplasma detection (Falagan‐Lotsch et al. 2015). For this, the TransDetect PCR Mycoplasma Detection Kit (TransGen Biotech) was used following the manufacturer's recommendations. Briefly, 40 µL of the FFFs media were individually incubated at 95°C for 10 min. Then, 2 µL of these samples were used as templates for the PCR reaction. The cycling parameters, which were programmed into a QIAquant 96 5 Plex thermocycler (Qiagen), consisted of an initial denaturation at 94°C for 10 min, followed by 35 cycles of denaturation (94°C, 30 s), annealing (60°C, 30 s), and extension (72°C, 30 s), ending with a final extension at 72°C for 5 min. The products were then cooled to 4°C and prepared for agarose gel electrophoresis using 1.5% agarose (Uniscience) gel containing 0.01% SYBR Safe DNA Gel Stain (Invitrogen), with 10 µL of the PCR product and 1 µL of loading buffer (Phoneutria). Electrophoresis was performed in an appropriate chamber with 1X TAE buffer for 40 min at 80 mV. Afterward, the gel was visualized using a UV transilluminator, and the results were interpreted based on the presence or absence of bands corresponding to the positive control provided by the kit.

### 2.12 Lipid Nanoparticle (LNP) Formulation

The LNP optimized for plasmidial DNA (pDNA) delivery employed in this study were previously developed and identified as effective carrier (Scalzo et al. 2022). In brief, an aqueous phase containing the pZsGreen‐N1 plasmid (Clontech Laboratories) was mixed with a lipid phase consisting of four components: (1) the ionizable lipid C12‐200; (2) the helper lipid 1,2‐dioleoyl‐sn‐glycero‐3‐phosphoethanolamine (DOPE; Avanti Polar Lipids); (3) cholesterol (Avanti Polar Lipids); and (4) 1,2‐dimyristoyl‐sn‐glycero‐3‐phosphoethanolamine‐N‐[methoxy(polyethylene glycol)‐2000] (ammonium salt) (C14‐PEG 2000; Avanti Polar Lipids). The two phases were combined using microfluidic mixing to formulate the LNPs, referred to as LNP4. Following formulation, the LNP4 suspension was transferred to a dialysis cassette with a 20 kDa molecular weight cut‐off (MWCO) and subjected to dialysis against PBS 1X for 2 h. Subsequently, the dialyzed LNPs were sterilized by filtration through a 0.22 µm PES membrane filter and then utilized in downstream in vitro experiments.

### 2.13 LNP4 Characterization

The physicochemical characterization of LNP4 was performed by assessing the hydrodynamic diameter, polydispersity index (PDI), and zeta potential of the nanoparticles. The hydrodynamic diameter and PDI were determined by dynamic light scattering (DLS). For this analysis, 10 µL of the LNP4 suspension were diluted in 990 µL of Milli‐Q ultrapure water and transferred to a suitable cuvette. Zeta potential measurements were obtained by evaluating the electrophoretic mobility of the particles. For this, 20 µL of the LNP4 formulation were diluted in 980 µL of 0.9% NaCl solution and analyzed in a specialized cuvette. All measurements were carried out using a Zetasizer Nano ZS‐90 instrument (Malvern Instruments, UK) under standard operating conditions.

### 2.14 Delivery and Expression of Exogenous Gene

To evaluate the efficiency of nanotransfection in fibroblast cell lines derived from Hyacinth Macaw feather follicles, 1 × 10⁴ cells were seeded into black, clear‐bottom 96‐well plates. Upon reaching approximately 80% confluence, cells were transfected with LNP4 nanoparticles encapsulating the pZsGreen‐N1 plasmid. The nanoformulated pDNA was administered at final concentrations of 0.4, and 0.8 µg per well. The culture medium was renewed every 24 h to ensure optimal cell viability and transgene expression. To monitor the kinetics of transgene expression, the intensity of green fluorescence protein (GFP) emission was measured at 24‐, 48‐, 72‐, 120‐, and 168‐h post‐transfection using the GFP filter set on the BioTek Cytation 5 Cell Imaging Multi‐Mode Reader (Agilent Technologies). In parallel, to assess potential cytotoxic effects resulting from nanotransfection, cell viability was quantified 72 h post‐exposure using the CellTiter‐Blue Cell Viability Assay (Promega), according to the manufacturer's instructions. Fluorescence intensity, indicative of metabolic activity, was measured using the same Cytation 5 platform. A negative control group, consisting of FFFs not exposed to LNP4, was included in all assays. All experimental conditions were performed in quintuplicate.

### 2.15 Statistical Analysis

Data are presented as mean ± SEM. Statistical significance between two groups was assessed using a two‐tailed unpaired Student's *t*‐test, performed with GraphPad Prism version 8.0.2. Data normality was evaluated using the Shapiro–Wilk test, and all datasets were confirmed to follow a normal distribution. Comparisons among multiple groups were analyzed using one‐way or two‐way ANOVA in combination with Tukey multiple comparisons test, as appropriate. A *p*‐value of less than 0.05 was considered statistically significant.

## 3 Results

### 3.1 Sample Collection, Cell Isolation, and Primary Culture

**Figure 1:**
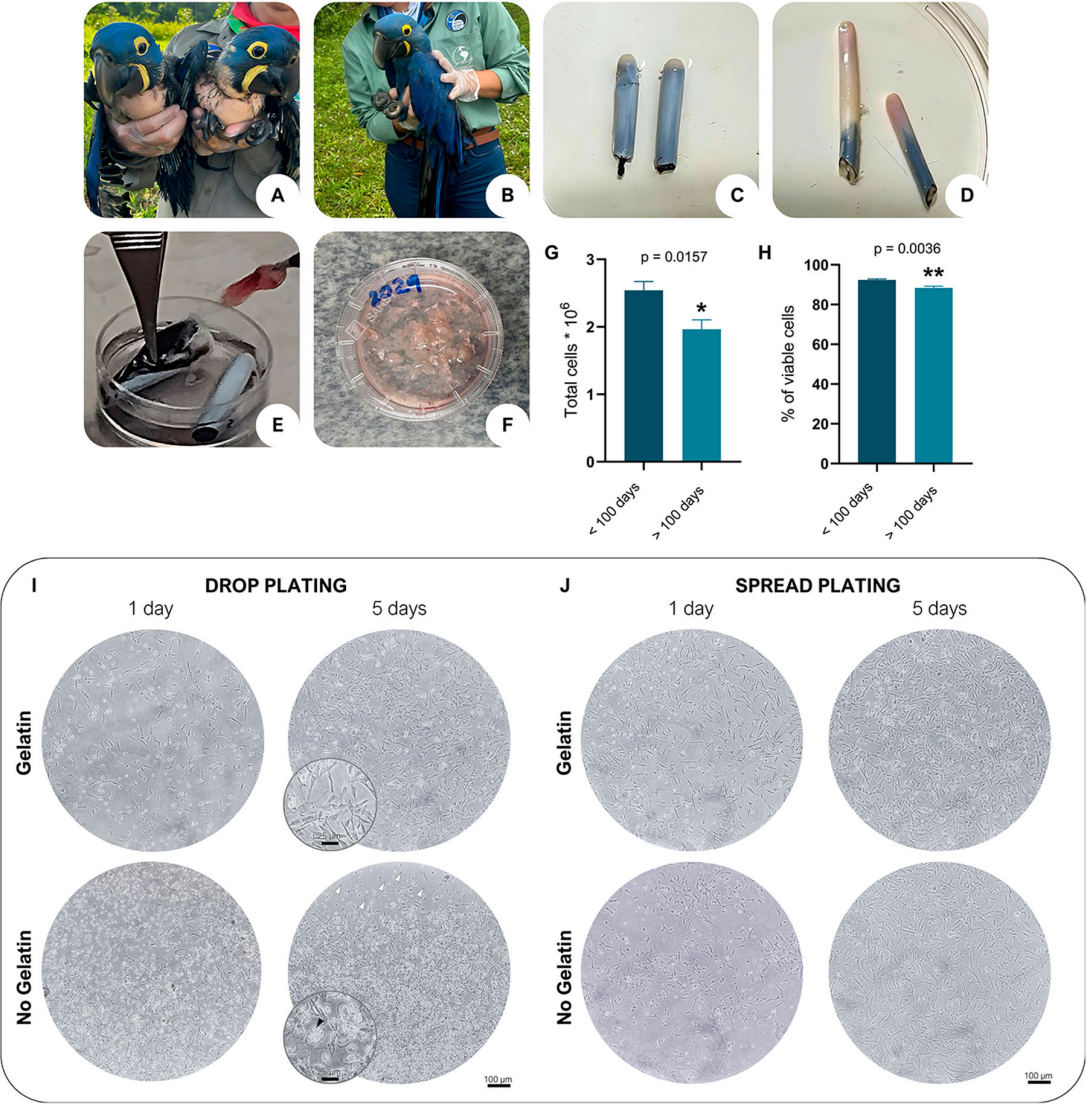
Feather collection, fibroblast isolation, and plating from Hyacinth Macaw (*A. hyacinthinus*). Representative size difference between nestlings younger than 100 days (A) and those older than 100 days (B), from which feather samples were obtained. Visual inspection of the feather calamus from younger (C) versus older birds (D), evidencing age‐related morphological differences. (E) Removal of the internal pulp from the calamus under aseptic conditions. (F) Mechanical processing of the extracted pulp. (G, H) Quantitative analyses showing significantly higher total cell yield and viability from feathers collected from birds under 100 days of age. Data are expressed as mean ± SD, and statistical comparisons were performed using the unpaired Student's *t*‐test (*p* < 0.05; *n* = 5 birds < 100 days; *n* = 6 birds > 100 days). (I, J) Hyacinth Macaw feather follicle fibroblasts (FFFs) plated under standard or droplet seeding conditions in gelatin‐coated or uncoated wells, evaluated at 24 h and 5 days post‐plating black arrows indicate morphological differences in the cells; white arrows highlight clustering patterns and unoccupied areas within the well. Scale bars: 100 µm; 25 µm in magnified views.

Due to the limited time between feather removal and processing, extractions were conducted within a single day to prevent significant cell loss. In both first and second year, feathers were collected from a similar number of Hyacinth Macaw, with 10 and 14 specimens, respectively. One feather from each wing was removed from the same region of each bird (Supporting Information S1: Figure S1A,B). The plumage was separated from the feather calamus, and after being washed in PBS with 2% of antibiotic‐antimycotic, the calamus were stored in DMEM‐F12 with 10% of FBS and 1% antibiotic‐antimycotic during transport (Supporting Information S1: Figure S1C,D). The age of the birds was determined based on field research conducted by the Arara Azul Institute. A marked age‐related difference was observed, with nestlings younger than 100 days (Figure 1A) presenting significantly smaller body size and less developed plumage when compared to individuals older than 100 days (Figure 1B), which display more advanced feather development.

A notable distinction observed during feather collection concerned the physical characteristics of the calamus. In younger birds (Figure 1C), the calamus retained a considerable amount of blue plumage, giving the impression of reduced pulp availability. In contrast, feathers collected from older individuals (Figure 1D) were more developed and exposed, with the calamus appearing more prominent and the pulp seemingly more abundant. The pulp was completely extracted from the calamus (Figure 1E) and processed individually for each bird (Figure 1F and Supporting Information S2: Figure S2). Comparative analysis of total cell yields between the two age groups revealed that nestlings younger than 100 days produced significantly higher average total cell counts (2.5 × 10⁶) compared to older birds (1.9 × 10⁶; *p* = 0.0157) (Figure 1G). Moreover, cell viability was also greater in the younger group, reaching 92% versus 88% in the older group (Figure 1H). These findings suggest that, when prioritizing nests for sampling, preference should be given to nests with younger Hyacinth Macaw nestlings.

In the first year, primary cultures were established in T25 cm² flasks without any prior surface coating, using a culture medium consisting of DMEM‐F12 supplemented with 10% FBS and 1% antibiotic‐antimycotic solution. After 4 months of continuous culture, only the FFFs samples from two individuals exhibited sufficient proliferation to allow passaging and subsequent cryopreservation. In the subsequent year, new experimental approaches were implemented to improve cell viability and proliferation rates. For this purpose, feather follicle cells were seeded into 24‐well plates, and both surface pre‐treatment protocols and cell seeding strategies were assessed. The majority of adherent cells observed within the first 2 h of culture exhibited an elongated, thin, spindle‐shaped morphology, consistent with fibroblasts. As shown in Figure 1J, 24 h following standard cell plating, wells pre‐treated with 0.2% gelatin displayed significantly enhanced and more homogeneous cell adhesion compared to untreated controls. However, this difference diminished over the first 5 days of culture, highlighting the relevance of gelatin pre‐treatment primarily during the initial adhesion phase. A distinct observation emerged regarding the drop cell seeding technique. This approach proved ineffective in gelatin‐coated wells, as the droplets rapidly dispersed, behaving similarly to standard plating conditions. Conversely, droplets applied to untreated wells initially showed the highest levels of cell adhesion among all tested conditions. Nonetheless, throughout the culture period, these cells exhibited subtle morphological alterations, including a less fusiform appearance and the presence of cytoplasmic vacuolization (Figure 1I). Moreover, the cells remained clustered, failing to spread across the culture surface. This clustering effect remained evident 5 days post‐plating, as illustrated in Figure 1I.

Therefore, although the droplet seeding method may initially promote superior adhesion of FFFs, it does not appear to be the most effective strategy for long‐term cultivation of these cells. In contrast, pre‐treatment of culture surfaces with gelatin represents a simple, cost‐effective approach that significantly enhances initial cell adhesion. Taken together, these findings support standard cell plating in gelatin‐coated wells as the most suitable method for establishing of Hyacinth Macaw FFF cultures.

### 3.2 Cryopreservation Evaluation and Post‐Thaw Survival Improvement

**Figure 2:**
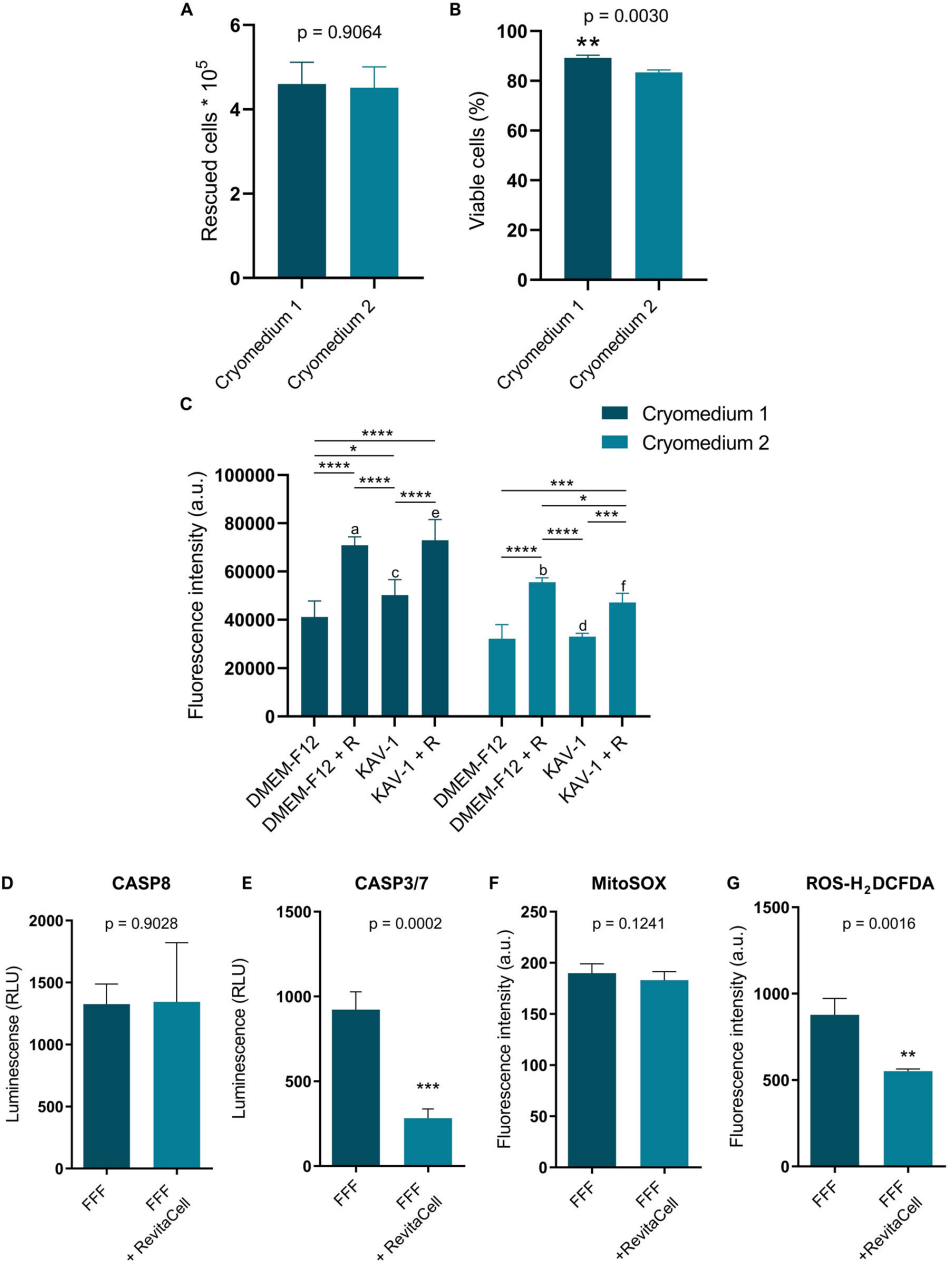
Post‐thaw cell recovery and viability analysis. (A) Total cell count recovered after thawing in Cryomedium 1 and Cryomedium 2. (B) Cell viability comparison reveals significantly higher viability in Cryomedium 1 compared to Cryomedium 2. Data are presented as mean ± SD; significance was calculated using Student's *t*‐test (*n* = 7/group). (C) Effect of RevitaCell supplementation on FFFs viability post‐thaw, in cells cultured in either Kuwana's Modified Avian Culture Medium‐1 (KAV‐1) or Dulbecco's modified Eagle medium/Ham's F12 (DMEM‐F12). Data represent means ± SD. Significance was calculated using a Tukey multiple comparisons test (*n* = 5). Asterisks denote statistical differences between cultivation conditions with and without RevitaCell supplementation within the same cryomedium (Cryomedium 1, *p = 0.0219; ****p < 0.0001. Cryomedium 2, *p = 0.0397; ***p < 0.0006; ****p < 0.0001). Different letters indicate statistically significant differences (*p* < 0.05) between cryomedia within the same culture condition. (D) Quantification of caspase‐8 activity in feather follicle fibroblasts (FFFs) cultured 24 h post‐thaw with RevitaCell supplementation. (E) Quantification of caspase‐3 activity in FFFs cultured 24 h post‐thaw with and without RevitaCell. Data represent means ± SD; significance was calculated using Student's *t*‐test (*n* = 6). Asterisks denote statistical differences (***p = 0.0002). (F) MitoSOX fluorescence measurement in post thaw FFF cultured with and without RevitaCell supplementation. (G) Quantification of total intracellular reactive oxygen species (ROS) in post‐thaw FFFs cultured with and without RevitaCell. Data represent means ± SD; significance was calculated using Student's *t*‐test (*n* = 6). Asterisks denote statistical differences (**p = 0.0016).

Upon reaching appropriate confluence (higher than 80%), the cells in passage #2 were prepared for cryopreservation (*n* = 12). To ensure good preservation condition of these difficult‐to‐obtain samples, two cryopreservation media were evaluated. A total of 5 × 10^5^ Hyacinth Macaw FFFs were frozen in Cryomedium 1 (DMEM‐F12‐based) and Cryomedium 2 (FBS‐based) (Supporting Information S2: Figure S2). The number of cells recovered post‐thawing was comparable between the two conditions (*p* = 0.9064), with an average recovery rate of approximately 90% (Figure 2A). However, Cryomedium 1 yielded significantly higher cell viability, with approximately 89% viable cells compared to 83% in Cryomedium 2 (Figure 2B). Although Hyacinth Macaw FFFs were efficiently recovered in both conditions, increased Trypan Blue uptake was observed in samples cryopreserved with Cryomedium 2, indicating a greater proportion of membrane‐compromised or non‐viable cells.

A post‐thaw culture period is essential to allow the progression of delayed‐onset cell death, enabling accurate assessment of cryoinjury and verification of the viability of the remaining cell population (Yu et al. 2024; Baust et al. 2022). Although immediate post‐thaw viability appeared satisfactory, a significant reduction in viable cells was observed within the first 24 h. To mitigate this loss, the effectiveness of RevitaCell, a supplement designed to buffer cellular stress responses during the post‐thaw recovery phase, was assessed. Figure 2C displays the results of a viability assay performed after thawing, in which RevitaCell was added during the cell washing step and maintained throughout the first 24 h of culture, using either DMEM‐F12 or KAV‐1 as the culture medium. Notably, within each cryopreservation medium group, all conditions treated with RevitaCell exhibited significantly higher cell viability 24 h post‐thaw compared to their respective untreated controls. Furthermore, significant intra‐medium differences were observed depending on the cryopreservation solution used (Figure 2C). Specifically, cells cultured in DMEM‐F12 supplemented with RevitaCell (*p* = 0.0005), in KAV‐1 without RevitaCell (*p* = 0.011), and in KAV‐1 with RevitaCell (*p* = 0.0045) showed significantly higher viability when previously cryopreserved in Cryomedium 1 compared to Cryomedium 2. These findings suggest that Cryomedium 1 is the most suitable formulation for the cryopreservation of Hyacinth Macaw FFFS, and that the inclusion of RevitaCell during the immediate post‐thaw period markedly improves cell survival and/or short‐term proliferation.

To explore the molecular mechanisms underlying this effect, we quantified the activity of caspase‐8 and caspase‐3, as well as measured mitochondrial superoxide production and total ROS levels in FFFs cultured post‐thaw with or without RevitaCell. While caspase‐8 activity remained unchanged (Figure 2D), a significant reduction in caspase‐3 activity (*p* < 0.05) was observed in FFFs cultured with RevitaCell (Figure 2E), suggesting that the supplementation may attenuate apoptosis, likely through modulation of the intrinsic apoptotic pathway. Furthermore, although mitochondrial superoxide levels remained unaffected (Figure 2F), total ROS levels were significantly higher (*p* < 0.01) in cells maintained without RevitaCell (Figure 2G), indicating that RevitaCell may also modulates cellular stress responses in FFFs, possibly by adjusting the balance between different ROS species or activating antioxidant mechanisms.

### 3.3 *A. Hyacinthinus* FFFs Behavior in Different Culture Media

**Figure 3:**
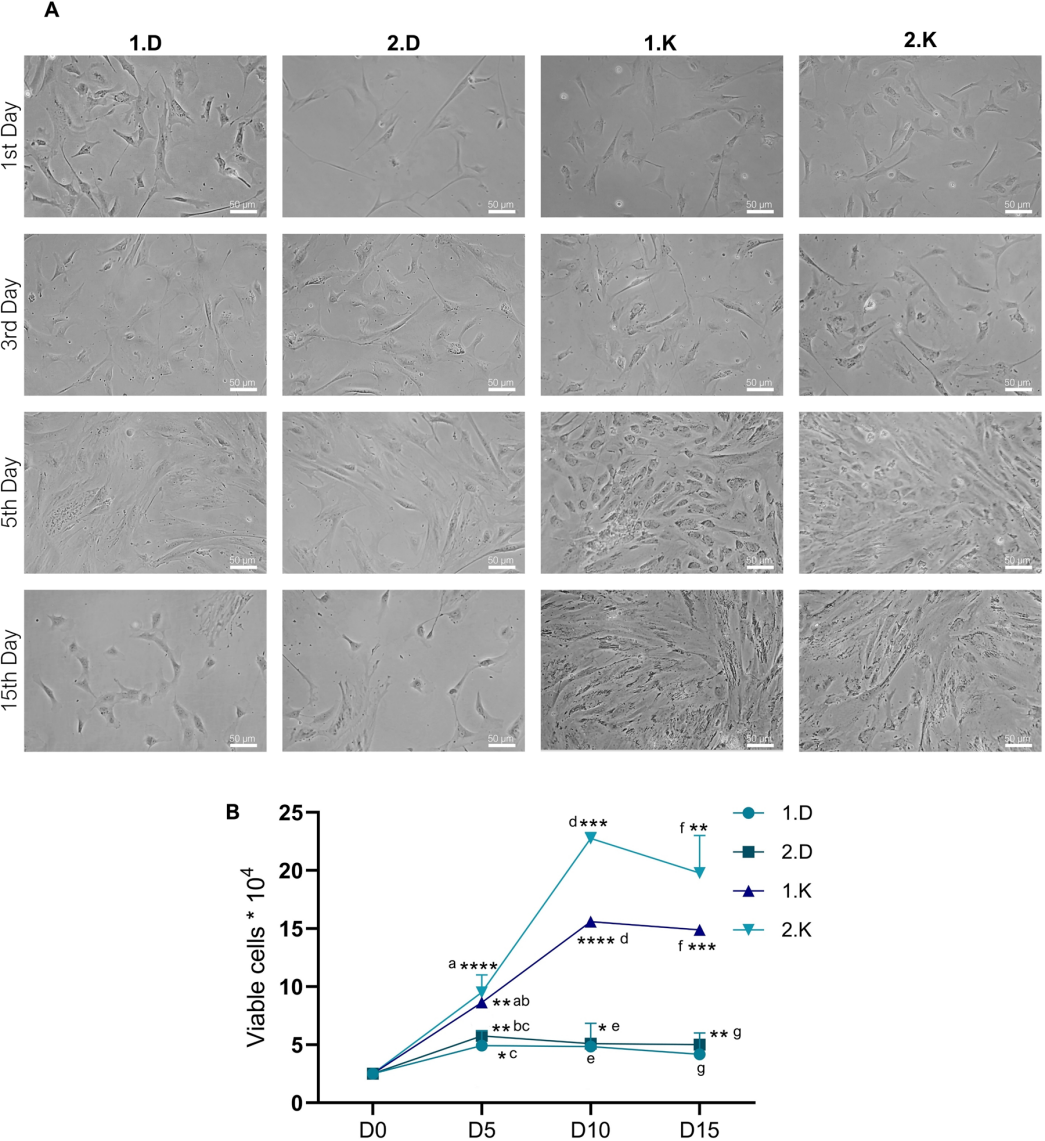
Effect of culture medium on the long‐term expansion of *A. hyacinthinus* feather follicle fibroblasts (FFFs). (A) Representative phase‐contrast images illustrating cell proliferation over a 15‐day period in Kuwana's Modified Avian Culture Medium‐1 (KAV‐1) and Dulbecco's Modified Eagle Medium/Ham's F12 (DMEM‐F12). FFFs cultured in KAV‐1 exhibited higher proliferation rates and reached greater confluence compared to those maintained in DMEM‐F12. The following group codes are indicated: 1.D—FFFs cryopreserved in Cryomedium 1 and cultured in DMEM‐F12; 2.D—FFFs cryopreserved in Cryomedium 2 and cultured in DMEM‐F12; 1.K—FFFs cryopreserved in Cryomedium 1 and cultured in KAV‐1; 2.K—FFFs cryopreserved in Cryomedium 2 and cultured in KAV‐1. Scale bar = 50 µm. (B) Quantitative assessment of cell counts on Days 5, 10, and 15 reveals significant differences in proliferation between media. Data represent means ± SD. Significance was calculated using a two‐way analysis of variance (ANOVA) in combination with a Tukey multiple comparisons test (*n* = 6/group). Asterisks denote significant difference between each time point and the initial time point (D0) within the same group (*p = 0.0290; **p < 0.0035; ***p < 0.0003; ****p < 0.0001). Different letters indicate statistically significant differences (*p* < 0.05) between groups at the same time point.

The decision to investigate KAV‐1 medium was based on previous studies suggesting its potential for superior performance in chicken fibroblast cultures (Katayama et al. 2021). Given that results from the initial 24 h post‐thaw cultures were inconclusive, we further examined the impact of different culture media on long‐term maintenance and proliferation of FFFs. A 15‐day culture evaluation was performed using both DMEM‐F12 and KAV‐1 media, with consideration of the origin of the cryopreservation medium, as illustrated in Figure 3A. FFFs exhibited significantly higher proliferation rates in KAV‐1 medium, achieving greater confluence by the end of the first 5 days of culture, irrespective of the cryomedium used. This pattern became more pronounced by Day 15 of culture (Figure 3A). To quantitatively assess cell proliferation, cell counts were performed at three time points: Days 5, 10, and 15, allowing for the generation of a growth curve (Figure 3B). Significant differences in cell numbers were observed across all time points, with FFFs cultured in KAV‐1 medium consistently exhibiting higher cell counts compared to those cultured in DMEM‐F12 medium. However, at Day 5, the cell count in Group 1.K was statistically similar to that of Group 2.D (*p* = 0.0882). Throughout the culture period, a significant increase in cell numbers (*p* < 0.05) was observed in Groups 1.K, 2.K, and 2.D on Days 5, 10, and 15 compared to Day 0 (Figure 3B). In contrast, Group 1.D did not exhibit significant cell proliferation beyond Day 10.

**Figure 4:**
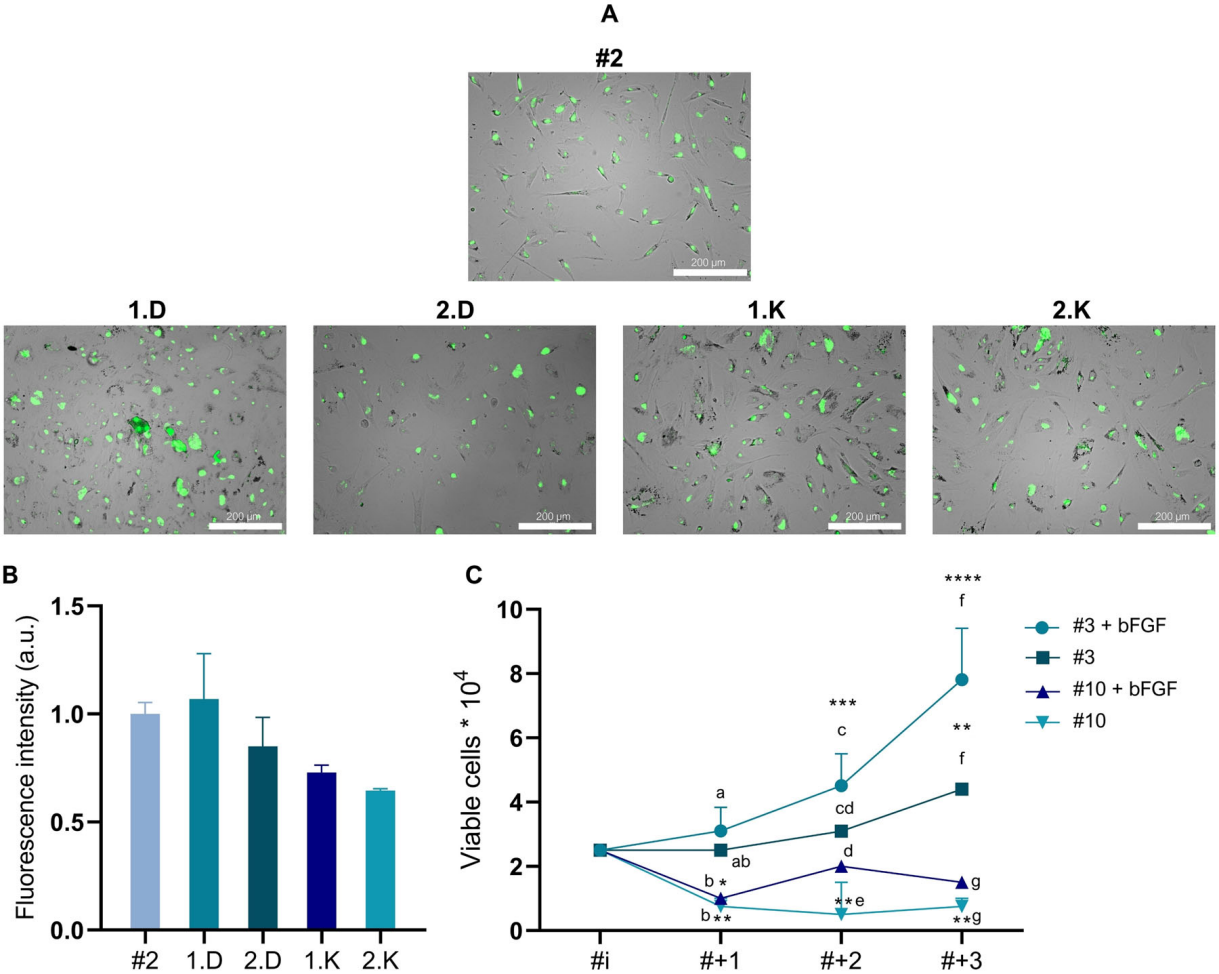
β‐Galactosidase (β‐Gal) activity and the proliferative effect of basic fibroblast growth factor (bFGF) in cultured *A. hyacinthinus* fibroblasts. (A) Representative fluorescence images of FFFs at Day 15 of culture, stained with CellEvent Senescence Green probe. Cells at early (passage #2) and late (passage #7) passages are shown. The following group codes are indicated: 1.D—FFFs cryopreserved in Cryomedium 1 and cultured in DMEM‐F12; 2.D—FFFs cryopreserved in Cryomedium 2 and cultured in DMEM‐F12; 1.K—FFFs cryopreserved in Cryomedium 1 and cultured in KAV‐1; 2.K—FFFs cryopreserved in Cryomedium 2 and cultured in KAV‐1. Scale bar = 200 µm. (B) Quantification of β‐Gal showing activity similar levels of fluorescence (*p* > 0.05) across all groups. (C) Effect of bFGF supplementation on the proliferation of FFFs cultured in KAV‐1 medium. The analysis was conducted using cells at passage 3 and passage 10 as initial points (#i), followed by three consecutive subcultures (# +1, # +2, and # +3). Quantitative cell counts demonstrated a significant proliferative enhancement in response to bFGF, particularly in early‐passage cells. Data represent means ± SD. Significance was calculated using a two‐way analysis of variance (ANOVA) in combination with a Tukey multiple comparisons test (*n* = 5/group). Asterisks denote statistically significant differences between each time point and the initial time point within the same group (*p < 0.0420; **p < 0.0057; ***p = 0.0005; ****p < 0.0001). Different letters indicate statistically significant differences (*p* < 0.05) between experimental conditions within the same subculture point.

To investigate the stagnation observed in cell proliferation of FFFs cultured in DMEM‐F12 and KAV‐1, a senescence assay was performed at the end of the 15‐day culture period, with cells at passage #7. For comparison, FFFs frozen at early passage (#2) of the same year were thawed. The results, as depicted in Figure 4A, revealed that all groups exhibited a similar pattern of SA‐β‐Gal staining. Quantitative analysis showed no significant differences in fluorescence levels between the cell groups (Figure 4B), indicating the absence of substantial senescence. This suggests that the observed lack of effective cell proliferation was likely not due to senescence, but rather possibly related to unmet biochemical requirements in the culture medium. To address the plateau in growth curves, an alternative approach was implemented by testing supplementation with bFGF. To quantify the effect of bFGF on cell proliferation, a growth curve was generated using FFFs at passage #10, with FFFs at passage #3 used for comparison. All cells were cultured in KAV‐1 medium for this experiment (Figure 4C). FFFs at passage #10 cultured without bFGF exhibited a significant reduction in cell numbers across the subsequent three passages (# +1, # +2, and # +3), reflecting a loss of cells. Notably, FFFs at passage #10 maintained with bFGF supplementation also showed a decrease in cell numbers at passage # +1, with no further growth observed thereafter (Figure 4C). As a result, no significant difference was observed between the initial number of seeded FFFs (#i) and the final cell count (# +3). Conversely, FFFs at passage #3, supplemented with bFGF, demonstrated significant proliferation at passage # +1 compared to the initial cell count (#i). By passage # +3, both the bFGF‐treated and non‐treated groups exhibited substantial growth relative to the initial seeding, indicating notable population expansion. Overall, no significant differences were observed between the KAV‐1 and KAV‐1 + bFGF conditions for FFFs at passages #3 and #10 at most time points. These findings suggest that bFGF supplementation at a concentration of 10 ng/mL was insufficient to sustain cell proliferation, especially in later passages. Passage number emerged as the primary factor influencing the behavior of Hyacinth Macaw FFFs, although this effect does not appear to be directly associated with cellular senescence.

### 3.4 FFF Cell Lines Characterization and Nanotransfection

**Figure 5:**
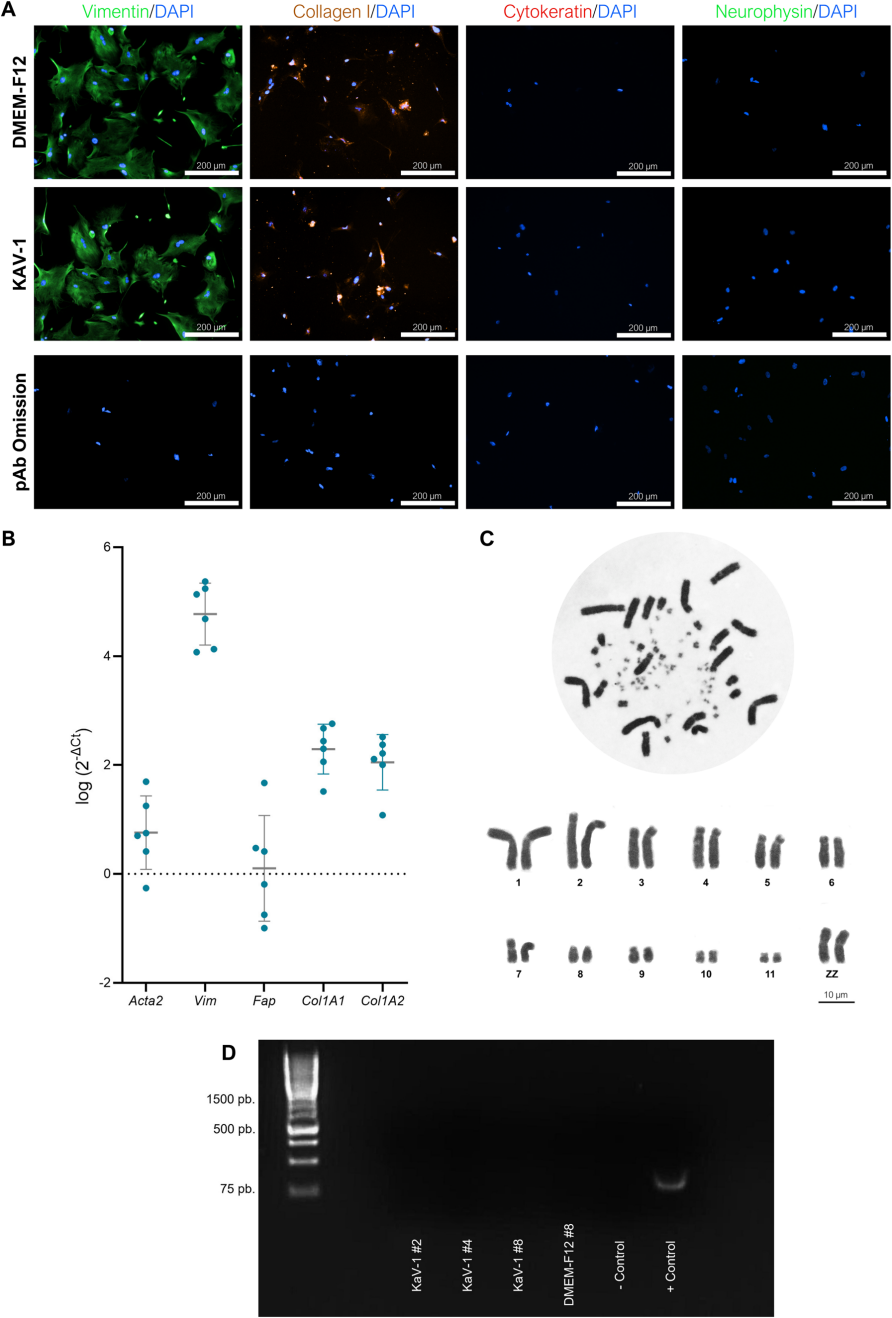
Characterization of feather follicle fibroblasts (FFFs) cell lines derived from *A. hyacinthinus*. (A) Immunofluorescence staining confirms the mesenchymal identity of the cultured FFFs. Vimentin expression (green, Alexa Fluor 488) was detected in cells maintained in both Kuwana's Modified Avian Culture Medium‐1 (KAV‐1) and Dulbecco's Modified Eagle Medium/Ham's F12 (DMEM‐F12), validating their fibroblastic phenotype. The cells also exhibited positive labeling for Type I collagen (red, Alexa Fluor 633) and were negative for cytokeratin (Alexa Fluor 546), a keratinocyte marker. Negative controls included omission of the primary antibodies and application of an unrelated antibody (anti‐neurophysin, Alexa Fluor 488). Scale bars = 200 µm. (B) Gene expression profiling confirms the presence of canonical fibroblast‐associated transcripts in the FFF lines. Data are presented as mean ± SD. Cycle threshold (Ct) values were normalized to TATA‐box Binding Protein (*Tbp*) gene. Average expression levels of target genes in FFF lines (*n* = 5/group) were calculate using 2^−ΔCt^ and expressed on log2 scale. (C) Representative karyotypic analysis of FFFs (passage #4) from a male *A. hyacinthinus*, showing a normal diploid chromosome number (2n = 70). Macrochromosomes are distinctly paired and arranged in a representative karyogram. Scale bar = 10 µm. (D) Mycoplasma testing was performed on FFFs at passages #2, #4, and #8 cultured in both DMEM‐F12 and KAV‐1 media. Gel electrophoresis was carried out using GeneRuler 1 kb Plus DNA Ladder (Invitrogen) in Lane 1. Sample lanes (3–6) were followed by a negative control (diethyl pyrocarbonate‐treated water, Lane 7) and a positive control (Lane 8). All tested samples were free of mycoplasma contamination.

Following the standardization of protocols for acquisition, isolation, cryopreservation, and optimal cultivation of FFFs, the next step focused on the characterization of the cell lines. To investigate the gene expression profile of the stabilized Hyacinth Macaw cells, we analyzed markers typically associated with fibroblasts using qPCR. As can be seen in Figure 5B, high transcript levels of *Acta2*, *Vim*, *Fap*, *Col1a1*, and *Col1a2* were detected. Furthermore, immunocytochemistry analysis supported the fibroblastic identity of these cells, demonstrating positive staining for vimentin (mesenchymal origin) and Type I collagen, and negative staining for cytokeratin (Figure 5A). These findings also confirm that the observed differences in cell morphology and behavior are attributable to the culture medium rather than to variations in cell type. Karyotypic analysis of FFFs confirmed the species‐specific diploid chromosome number (2n = 70), comprising 24 macrochromosomes and 46 microchromosomes. The macrochromosomes were paired and aligned, showing 3 metacentric pairs (1, 7, and 10), 5 submetacentric pairs (5, 6, 8, 9, and 11), and 3 acrocentric pairs (2, 3, and 4), consistent with previous reports (de Oliveira Furo et al. 2015; Lunardi et al. 2003). Specifically, Figure 5C depicts the karyotype of a male individual, as evidenced by the presence of two morphologically identical metacentric sex chromosomes, corresponding to the Z chromosomes of the species. Mycoplasma detection tests were conducted for FFF cell lines from different passages (#2, #4, and #8) in both KAV‐1 and DMEM‐F12 media, ensuring that this cell line is free from mycoplasma contamination (Figure 5D).

**Figure 6:**
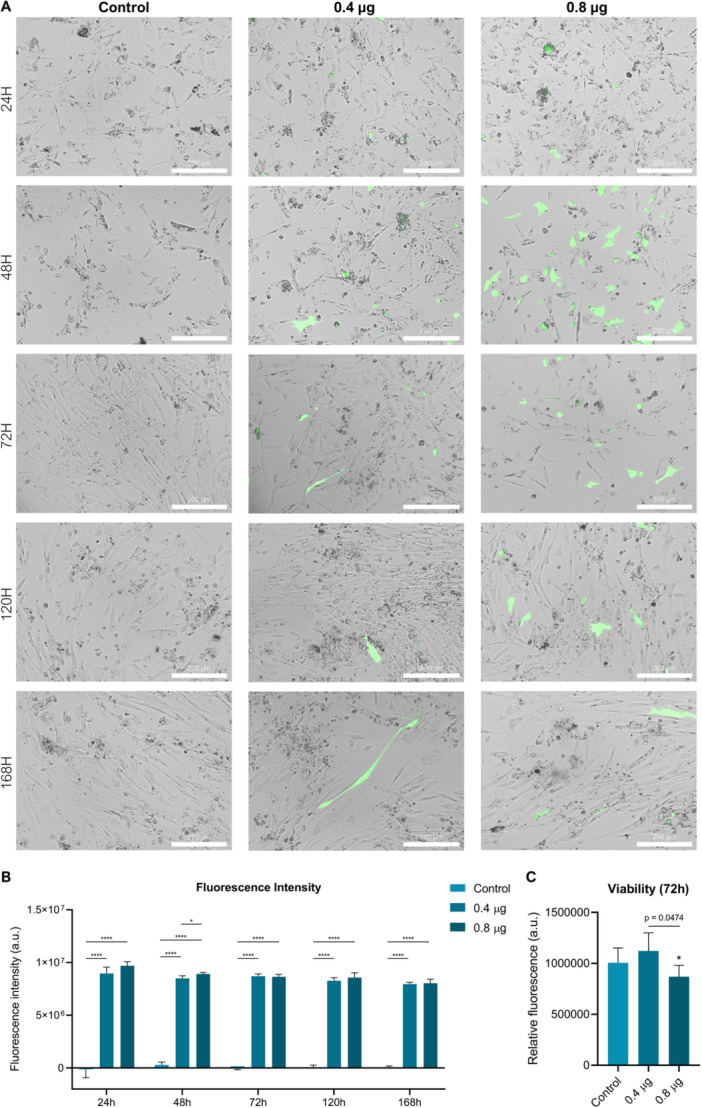
Transfection efficiency of *A. hyacinthinus* feather follicle fibroblasts (FFFs) cell lines using lipid nanoparticles. (A) Representative bright‐field and fluorescence images showing green fluorescent protein (GFP) expression in FFFs at 24, 48, 72, 120, and 168 h after exposure to LNP4‐pZsGreen‐N1 complexes. Scale bar = 200 µm. (B) Quantification of GFP fluorescence intensity over time reveals sustained transgene expression in FFFs up to 168 h post‐transfection. Data represent means ± SD. Significance was calculated using a two‐way analysis of variance (ANOVA) in combination with a Tukey multiple comparisons test (*n* = 5/group). Asterisks denote statistically significant differences between treatment (*p = 0.0343; ****p < 0.0001). (C) Quantification of cell viability after nanotransfection. Data represent means ± SD. Significance was calculated using a one‐way ANOVA in combination with a Tukey multiple comparisons test (*n* = 5/group). Asterisks denote statistically significant differences between treatments.

In this study, LNPs were prepared using a mixture of an ionizable lipid, a helper lipid, cholesterol, and a lipid‐PEG in a previously optimized molar ratio (Scalzo et al. 2022). To characterize the LNP formulation, we evaluated the size, PDI, and zeta potential. DLS measurements showed that pDNA‐LNP complexes had a diameter between 150 and 170 nm, with a PDI below 0.2. Additionally, zeta potential measurements showed a value of −8.71 ± 3.52, indicating a slightly negative surface charge. To evaluate the transfection potential of FFF cell lines using LNPs, we assessed their capacity to express the GFP reporter gene following exposure to LNP4‐pZsGreen‐N1 complexes. As shown in Figure 6, cells were treated with 0.4 and 0.8 µg of the complex, and GFP fluorescence was monitored. Efficient pDNA delivery was evidenced by detectable GFP expression as early as 24‐h post‐exposure at both concentrations (Figure 6A). A significant increase in the number of GFP‐positive cells was observed at 48 h in cultures treated with 0.8 µg, demonstrating a concentration‐dependent enhancement in initial transgene expression (Figure 6A,B). However, this difference was not maintained over time, with comparable levels of fluorescence observed across both treatment groups in subsequent analyses (Figure 6B). Notably, LNP4‐pZsGreen‐N1 exposure resulted in minimal cytotoxicity in FFFs, with cell viability remaining comparable to the untreated control at the tested concentration (Figure 6C). Additionally, sustained GFP expression was observed in FFSs up to 168 h, with no significant difference in fluorescence intensity between the two treatment groups (Figure 6B). These results demonstrate that Hyacinth Macaw FFFs are amenable to transfection via LPN and are capable of sustaining exogenous gene expression driven by CMV promoter.

## 4 Discussion

Embryonic and adult fibroblasts are extensively employed in iPSC generation owing to their high availability, robust in vitro proliferation, permissive epigenetic landscape, and compatibility with both viral and non‐viral gene delivery approaches. Their suitability for reprogramming has been demonstrated in multiple studies reporting the derivation of avian iPSCs from these cells (Katayama et al. 2018, 2022; Lu et al. 2012, 2015; Nogueira et al. 2024). However, within the context of conservation, it is imperative to adopt sampling strategies that minimize harm to donor organisms. In this regard, dermal fibroblasts isolated from feather follicles offer a minimally invasive alternative. These cells can be obtained by plucking feathers from the primary and secondary wing remiges, a method that causes negligible impact on the health and behavior of the animals (Kim et el. 2017; Kroglund et al. 2022).

Long‐term studies on the biology, management, and conservation of the Hyacinth Macaw have been conducted since the 1990s (Guedes and Harper 1995; Guedes 2004; Guedes 2009; dos Santos Ferreira et al. 2023; Vilaça et al. 2024) Nest monitoring represents the main strategy for data collection, as breeding pairs show fidelity to nesting sites (Miyaki et al. 1995). Visits are scheduled at intervals of up to 10 days and are carried out swiftly and carefully, following standardized techniques that minimize disturbance and stress. Importantly, over 36 years of fieldwork have shown no evidence of nest abandonment associated with this method (Guedes and Seixas 2002; Guedes 2004). During egg and chick handling, measures are consistently taken to minimize stress for both nestlings and adults. Parents of younger chicks (up to ~70 days old) typically remain perched nearby in a vigilant posture and promptly return to the nest after procedures, while interventions in older chicks often coincide with natural parental absences, further reducing the likelihood of disturbance. These long‐standing practices are in accordance with internationally accepted avian welfare and conservation guidelines, providing robust ethical justification for their application (de de Andrade Ramalho et al. 2024; Guedes et al. 2022). Therefore, research, management, and conservation efforts on the Hyacinth Macaw in Brazil have become a reference not only for this species but also for other psittacids worldwide.

Due to sampling time limits and logistical constraints, investigating the Hyacinth Macaw required prioritizing nests for sample collection. Therefore, making the correct choice of nestlings in the field is crucial. Initially, based solely on visual assessment of the feathers from young nestlings, where much of the plumage remained inside the feather calamus, it appeared that there was insufficient fleshy pulp for cell acquisition. However, upon sample processing, it was revealed that prioritizing nests with younger macaws in‐deed proved more advantageous due to higher cellular yield and viability. A notable modification introduced to the feather removal process in the second year involved the application of local ice for 2 min, which resulted in lower rates of bleeding and local tenderness.

The substantial challenges encountered during primary cell culture in the first year highlight the critical need for species‐specific standardization in the collection and cultivation of biological material from elusive wildlife. Protocols routinely applied to domestic species frequently fail to address the distinct physiological and cellular demands of exotic taxa (Dicks et al. 2021). In the second year, three key methodological modifications markedly enhanced culture outcomes. First, Hyacinth Macaw cells were seeded onto a surface area better matched to the initial yield, promoting optimal cell‐to‐cell contact and improving survival rates (Yao et al. 2013). Second, pre‐treatment of culture plates with gelatin significantly improved fibroblast adhesion and selective enrichment (Almazloum and Khalil 2023; Garvin and Katwa 2023). Third, post‐thaw proliferation was notably enhanced by the use of KAV‐1 medium, consistent with reports on avian fibroblast culture, particularly in chicken models (Katayama et al. 2021). Collectively, these adjustments underscore the importance of protocol optimization for successful *ex vivo* expansion of avian cells from non‐model organisms.

Following the optimization of culture conditions, sufficient cell proliferation was achieved in the second year of collection, allowing for the successful passage and subsequent cryopreservation of Hyacinth Macaw FFFs. One of the most commonly used cryopreservation media combinations is FBS and DMSO, which effectively maintains cell recovery and viability for most mammalian cell lines, with optimal outcomes reported using 5% or 10% DMSO in conjunction with FBS (Zhao et al. 2001). DMSO is essential for stabilizing cell proteins and membranes, preventing intracellular ice formation, and enhancing cell survival during freezing, and some studies also support the use of conventional complete culture media with DMSO for this purpose (Di Bella et al. 2021; Lee et al. 2012).

In this study, a significant finding was the superior performance of Cryomedium 1, based on DMEM‐F12, when compared to Cryomedium 2, the FBS‐based medium. Moreover, cells frozen in Cryomedium 1 demonstrated superior performance during the post‐thaw phase, further validating its effectiveness for the cryopreservation Hyacinth Macaw FFFs. In mammalian and avian cell culture, FBS is widely employed as a non‐permeating cryoprotective agent due to its ability to increase medium viscosity and mitigate oxidative stress (Harper et al. 2011; Tonus et al. 2016; S. Park et al. 2018; Yang et al. 2020; Chaipipat et al. 2021; Katayama et al. 2022). However, reducing the FBS concentration in cryopreservation solutions, from conventionally high levels (e.g., 90%) to more moderate levels (e.g., 35%), may confer advantages. Lower concentrations of FBS are associated with reduced cytotoxicity, thereby improving post‐thaw cell viability and recovery rates. Moreover, they better preserve cellular functionality and phenotype, which is critical for downstream applications (López et al. 2016). Optimizing the balance between permeating and non‐permeating cryoprotectants, such as FBS, is essential for maximizing cell survival and functional recovery (Ha et al. 2016). Additionally, the use of lower FBS concentrations represents a cost‐effective strategy, offering adequate cryoprotection while significantly reducing expenses, an especially relevant consideration in large‐scale crybanking efforts (Zalomova and Fesenko 2024). In this context, further studies are required to systematically test and optimize FBS concentrations below 35%, ensuring both efficiency and cost‐effectiveness for the establishment of Hyacinth Macaw biobanks. Notably, although the choice of cryomedium exerts a pronounced influence during the early post‐thaw phase, our findings indicate that, over the medium to long term, the culture medium becomes the predominant factor shaping cellular behavior and stability in Hyacinth Macaw FFFs.

The dysregulation of biochemical pathways following cryopreservation can trigger a phenomenon known as cryopreservation‐induced delayed‐onset cell death (CIDOCD), characterized by cellular demise occurring hours to days after thawing. This delayed cell death can represent a critical bottleneck in the field of cell and tissue biobanking, substantially compromising long‐term viability and functional utility of preserved biological samples (Baust et al. 2015). In the present study, we observed that the supplementation of culture medium with RevitaCell significantly improved the post‐thaw recovery of FFFs, suggesting a potential strategy to mitigate CIDOCD‐related losses. RevitaCell is composed of a selective ROCK inhibitor combined with antioxidant and free radical scavenging molecules (Y.‐H. Chen and Pruett‐Miller 2018; Sakai et al. 2023) The improved cellular outcomes observed in our study likely result from the synergistic action of these components. The ROCK inhibitor may have modulated key intracellular signaling pathways involved in FFFs survival and cytoskeletal integrity, thereby reducing apoptosis through the inhibition of pro‐apoptotic mechanisms within the intrinsic pathway. In parallel, the antioxidant components likely attenuated oxidative stress, which is known to exacerbate post‐thaw cell damage (Leão et al. 2021). These findings emphasize the importance of post‐thaw culture conditions in promoting Hyacinth Macaw FFFs recovery and viability. Moreover, they demonstrate the potential of targeted supplementation strategies, such as the use of RevitaCell, to enhance cellular resilience and functional stability following cryopreservation of avian cells, contributing to the refinement of protocols for ex situ conservation of endangered species.

With a reliable cryopreservation protocol in place, subsequent experiments were designed to evaluate the effects of culture media on cell proliferation. Cultivation in DMEM‐F12 led to stagnant growth of FFFs, similar to observations from the first year of collection when cells were maintained in this medium for extended periods. In contrast, the KAV‐1 resulted in significantly enhanced cell proliferation rate. Although the growth curve began to decline and plateau by the 15th day, FFFs responded more effectively to KAV‐1. This enhanced performance is likely linked to the optimized formulation of KAV‐1, which may reduce cellular stress and provide more robust metabolic support during in vitro expansion. These observations align with findings by Katayama et al. (2022), who successfully cultured fibroblasts derived from endangered avian species in KAV‐1 medium following reprogramming. Furthermore, the improved growth of embryonic chicken fibroblasts in KAV‐1, when compared to DMEM and Medium 199, has also been documented (Katayama et al. 2018). KAV‐1 is an MEM‐Alpha–based culture medium. Unlike DMEM‐F12, MEM‐Alpha contains ascorbic acid, a vitamin that not only plays a critical role in collagen biosynthesis, but also have antioxidant properties. Based on this difference, we hypothesize that KAV‐1 may provide a more favorable environment Hyacinth Macaw FFFs growth and viability (Amano et al. 2007). In addition, MEM‐Alpha presents a higher concentration of lipoic acid, a compound associated with antioxidant properties, which could further contribute to cellular protection. This compound is also related to the regeneration of other antioxidants, such as ascorbic acid (Capece et al. 2022). Another distinctive feature of MEM‐Alpha is the presence of nucleosides in its formulation, supporting DNA biosynthesis and potentially enhancing cell proliferation (Tang et al. 2018). An additional consideration is that serum derived from phylogenetically closer species may provide growth factors, hormones, and extracellular matrix components that are more physiologically compatible with avian cells (Kim et al. 2021; Katayama et al. 2021). In this context, the enhanced proliferation of Hyacinth Macaw FFF observed in KAV‐1 medium, which includes chicken serum, reinforces the idea that avian‐derived serum supplements can more accurately recapitulate the natural microenvironment of psittacid cells. Although large‐scale implementation of chicken serum is limited by availability and standardization challenges, even modest supplementation may constitute a valuable strategy to improve the culture performance of non‐domesticated or conservation‐relevant avian cells. Our results further point to the need for mechanistic studies to elucidate the molecular basis underlying these beneficial effects.

To assess why the fibroblasts were not proliferating as expected, a senescence test was conducted. Activation of β‐galactosidase, a common biomarker for mammals’ senescent cells, was used (Zhai et al. 2019). The lack of significant differences in SA‐β‐Gal activity between groups cultured in DMEM‐F12 and KAV‐1 at passage #10, and passage #2 suggests two possibilities. First, considering FFFs at passage #2 as non‐aging cells, it implies that the experimental cells at passage #10 did not reach senescence, and their low proliferation was due to unmet demands. Alternatively, if passage #2 FFFs were senescent, then all other cells were senescent as well, indicating that some factor was inducing aging either during the primary culture or immediately post‐thaw. Clarifying the true senescence status of early‐passage FFFs will require a more comprehensive understanding of avian fibroblast aging and its molecular markers. This irreversible arrest of cell division can occur due to various forms of senescence (Campisi and d'Adda di Fagagna 2007), such as replicative senescence (Hayflick and Moorhead 1961) stress‐induced premature senescence (Toussaint et al. 2000), and oxidative stress‐induced senescence (Chen et al. 1998), leading to changes in cell function, morphology, and gene expression. Further investigation into senescence‐associated pathways, including p53/p21 and p16 signaling, as well as associated cellular changes (Di Leonardo et al. 1994; Serrano et al. 1997; Campisi and d'Adda di Fagagna 2007; Chandra et al. 2015), will be important to elucidate the mechanisms involved and refine culture strategies.

To test the hypothesis that the limited proliferative capacity observed in FFFs was due to insufficient trophic support in the culture medium, we supplemented the cultures with bFGF. This growth factor is widely recognized as essential for the expansion and maintenance of animal fibroblasts, exerting potent mitogenic effects and promoting extracellular matrix production (Floege et al. 1992; Ogata et al. 2001; C. X. Liu et al. 2014). It also modulates the release of heparan sulfate proteoglycans, which are key regulators of growth factor signaling (Buczek‐Thomas and Nugent 1999). Given these well‐established functions, the addition of bFGF was expected to overcome the apparent proliferation plateau, provided that the cells had not reached senescence. However, the supplementation failed to produce an increase in cell proliferation when compared to unsupplemented controls within the same passages. This indicates that bFGF, under the tested conditions, was insufficient to restore or enhance proliferative activity in Hyacinth Macaw FFFs. One plausible explanation is that the dosage applied was suboptimal for this species. Although FGF receptors are evolutionarily conserved across vertebrates (Goetz et al. 2009; Goetz and Mohammadi 2013), interspecies differences in receptor affinity and intracellular signaling efficiency may compromise the biological activity of human recombinant bFGF in Hyacinth Macaw cells. These findings suggest that the use of human‐derived growth factors in non‐model species requires empirical validation, and future dose‐response studies are warranted to clarify the responsiveness of FFFs to bFGF.

Recent efforts have focused on inducing pluripotency in avian fibroblast, primarily through the use of viral vectors and transposon‐based systems (Nogueira et al. 2024). While such strategies have yielded valuable insights, the generation of iPSCs in endangered avian species demands efficient and biosafe alternatives that avoid permanent genomic integration. In this context, LNPs have emerged as a compelling non‐viral nucleic acid delivery platform, offering several key advantages, including ease of formulation, high encapsulation efficiency, cellular uptake proficiency, endosomal escape capability, and low immunogenicity (Zhang et al. 2024; Scalzo et al. 2022). The ionizable LNP4 formulation, in particular, has demonstrated efficacy in facilitating heterologous gene expression in mammalian cells that are typically refractory to transfection and exhibit low proliferation rates (Scalzo et al. 2022). Based on these characteristics, we explored its application in Hyacinth Macaw FFFs as an initial proof of concept. To our knowledge, this is the first study to report successful gene delivery using LNPs in avian cells, an important step that not only expands the methodological toolkit available for working with non‐model bird species, but also sets the stage for non‐integrative reprogramming strategies in conservation biology. Building upon this advance, LNP platforms platform holds significant promise for future RNA‐based applications, which circumvent the requirement for nuclear entry and enable immediate cytosolic translation, an especially attractive feature when working with rare or sensitive cell types (Yoshioka and Dowdy 2017; Hou et al. 2021) Although the derivation of avian iPSCs remains a complex and stepwise endeavor, requiring rigorous optimization at each stage, the data presented here represent a foundational advancement.

While studies will continue, the first step towards developing biotechnology for the conservation of the Hyacinth Macaw has been taken by successfully establishing and standardizing the acquisition and maintenance of primary cell lines. The fibroblast cultures derived from feather follicles exhibited a normal karyotype, consistent with cytogenetic profiles previously reported for the species (Lunardi et al. 2003; de Oliveira Furo et al. 2015). This chromosomal stability reinforces the experimental reproducibility and confirms the genetic integrity of our cell lines—an essential prerequisite for downstream applications involving genome editing, reprogramming, or biobanking. Furthermore, rigorous quality control measures were implemented to ensure the biosafety of the cultures. Notably, the established cell lines were confirmed to be free from mycoplasma contamination—an often‐underestimated issue that can significantly compromise experimental outcomes by altering cellular physiology and gene expression (Lincoln and Gabridge 1998). Given the serious implications of mycoplasma contamination for both autologous and heterologous cell‐based therapies, these cell lines will be continuously monitored for contamination, especially since mycoplasma is not frequently reported in early passages (Floege et al. 1992).

In summary, the *A. hyacinthinus* cell lines established in this study have been thoroughly characterized, exhibiting a stable karyotype, high viability, absence of microbial contamination, and successful transfection using LNPs. Regarding its collection, isolation, and cultivation, it is clear that priority should be given to collecting from nests with younger animals under 100 days of age. Freezing *A. hyacinthinus* FFFs in 55% DMEM‐F12 with 35% FBS and 10% DMSO, followed by thawing with RevitaCell and cultivation in pre‐treated gelatin plates with KAV‐1 medium, proved comparatively more efficient and advantageous. Although the number of nestlings included may not fully capture the species’ genetic diversity, and broader sampling across different populations or related species will be important in future studies to enhance genetic representation, our findings emphasize the potential of these cell lines for biobank formation and their utility as an experimental model in projects aiming to develop iPSCs and PGCs for wildlife conservation and recovery programs.

## Author Contributions

Conceptualization: Iara Pastor Martins Nogueira, and Samyra Maria dos Santos Nassif Lacerda. Methodology: Iara Pastor Martins Nogueira, Rachel Castro Teixeira‐Santos, Gustavo Caldeira Cotta, Wanderson Valente, John Lennon de Paiva Coimbra, Heloísa Athaydes Seabra Ferreira, Pedro Pires Goulart Guimarães, Anderson Kenedy Santos, Fernanda Mussi Fontoura, Kefany Rodrigues de Andrade Ramalho, Neiva Maria Robaldo Guedes, and Samyra Maria dos Santos Nassif Lacerda. Formal analysis: Iara Pastor Martins Nogueira, Rachel Castro Teixeira‐Santos, Gustavo Caldeira Cotta, Wanderson Valente, John Lennon de Paiva Coimbra, and Samyra Maria dos Santos Nassif Lacerda. Investigation: Iara Pastor Martins Nogueira, Rachel Castro Teixeira‐Santos, Gustavo Caldeira Cotta, Wanderson Valente, John Lennon de Paiva Coimbra, and Samyra Maria dos Santos Nassif Lacerda. Resources: Neiva Maria Robaldo Guedes and Samyra Maria dos Santos Nassif Lacerda. Data curation: Iara Pastor Martins Nogueira, Rachel Castro Teixeira‐Santos, Gustavo Caldeira Cotta, Wanderson Valente, John Lennon de Paiva Coimbra, and Samyra Maria dos Santos Nassif Lacerda. Writing—original draft preparation: Iara Pastor Martins Nogueira, Rachel Castro Teixeira‐Santos, and Gustavo Caldeira Cotta. Writing—review and editing: Iara Pastor Martins Nogueira, Rachel Castro Teixeira‐Santos, Gustavo Caldeira Cotta, Wanderson Valente, John Lennon de Paiva Coimbra, and Samyra Maria dos Santos Nassif Lacerda. Visualization: Iara Pastor Martins Nogueira, Rachel Castro Teixeira‐Santos, Gustavo Caldeira Cotta, Wanderson Valente, John Lennon de Paiva Coimbra, and Samyra Maria dos Santos Nassif Lacerda. Supervision: Samyra Maria dos Santos Nassif Lacerda. Project administration: Samyra Maria dos Santos Nassif Lacerda. Funding acquisition: Neiva Maria Robaldo Guedes and Samyra Maria dos Santos Nassif Lacerda. All authors have read and agreed to the published version of the manuscript.

## Conflicts of Interest

The authors declare no conflicts of interest.

## Supporting information


**Supplementary Figure 1.** Sequential steps for in‐field collection of Hyacinth Macaw feathers from nestlings. The procedure begins with the selection of an optimal feather (A), followed by careful extraction of the calamus (B). The calamus is then separated from the feather and placed in a centrifuge tube for washing (C). Finally, the feather samples are immersed in culture medium for transport under controlled conditions (D).

**Supplementary Figure 2.** Overview of *A. hyacinthinus* feather follicle fibroblasts (FFFs) isolation and cryopreservation workflow.

## Data Availability

The data that support the findings of this study are available from the corresponding author upon reasonable request.

## References

[cbin70089-bib-0001] Allgayer, M. C. , N. M. R. Guedes , C. Chiminazzo , M. Cziulik , and T. A. Weimer . 2009. “Clinical Pathology and Parasitologic Evaluation of Free‐Living Nestlings of the Hyacinth Macaw (*Anodorhynchus hyacinthinus*).” Journal of Wildlife Diseases 45, no. 4: 972–981. 10.7589/0090-3558-45.4.972. 19901373

[cbin70089-bib-0002] Almazloum, A. , and H. Khalil . 2023. “Isolation of Adult Mouse Cardiac Fibroblasts.” Current Protocols 3, no. 7: e840. 10.1002/cpz1.840. 37439518

[cbin70089-bib-0003] Amano, S. , Y. Ogura , N. Akutsu , and T. Nishiyama . 2007. “Quantitative Analysis of the Synthesis and Secretion of Type VII Collagen in Cultured Human Dermal Fibroblasts With a Sensitive Sandwich Enzyme‐Linked Immunoassay.” Experimental Dermatology 16, no. 2: 151–155. 10.1111/j.1600-0625.2006.00514.x. 17222230

[cbin70089-bib-0004] de Andrade Ramalho, K. R. , F. M. Fontoura , and N. M. R. Guedes . 2024. “First Record of Free‐Living Hyacinth Macaw (*Anodorhynchus hyacinthinus*) Eggs Hatching Using Camera Traps in Southern Pantanal, Brazil.” Ornithology Research 33, no. 1: 6. 10.1007/s43388-024-00207-y.

[cbin70089-bib-0005] Baust, J. M. , W. L. Corwin , R. VanBuskirk , and J. G. Baust . 2015. Biobanking: The Future of Cell Preservation Strategies, 37–53. 10.1007/978-3-319-20579-3_4. 26420612

[cbin70089-bib-0006] Baust, J. M. , K. K. Snyder , R. G. Van Buskirk , and J. G. Baust . 2022. “Assessment of the Impact of Post‐Thaw Stress Pathway Modulation on Cell Recovery Following Cryopreservation in a Hematopoietic Progenitor Cell Model.” Cells 11, no. 2: 278. 10.3390/cells11020278. 35053394 PMC8773610

[cbin70089-bib-0007] Di Bella, S. , V. Cannella , F. Mira , et al. 2021. “The Effect of a 7 Year‐Long Cryopreservation on Stemness Features of Canine Adipose‐Derived Mesenchymal Stem Cells (cAD‐MSC).” Animals : An Open Access Journal from MDPI 11, no. 6: 1755. 10.3390/ani11061755. 34208255 PMC8230844

[cbin70089-bib-0008] Buczek‐Thomas, J. A. , and M. A. Nugent . 1999. “Elastase‐Mediated Release of Heparan Sulfate Proteoglycans From Pulmonary Fibroblast Cultures.” Journal of Biological Chemistry 274, no. 35: 25167–25172. 10.1074/jbc.274.35.25167. 10455199

[cbin70089-bib-0009] Campisi, J. , and F. d'Adda di Fagagna . 2007. “Cellular Senescence: When Bad Things Happen to Good Cells.” Nature Reviews Molecular Cell Biology 8, no. 9: 729–740. 10.1038/nrm2233. 17667954

[cbin70089-bib-0010] Capece, U. , S. Moffa , I. Improta , et al. 2022. “Alpha‐Lipoic Acid and Glucose Metabolism: A Comprehensive Update on Biochemical and Therapeutic Features.” Nutrients 15, no. 1: 18. 10.3390/nu15010018. 36615676 PMC9824456

[cbin70089-bib-0011] Cardoso, C. A. , L. C. B. Motta , V. C. de Oliveira , and D. S. Martins . 2020. “Somatic Feather Follicle Cell Culture of the Gallus Domesticus Species for Creating a Wild Bird Genetic Resource Bank.” Animal Reproduction 17, no. 3: e20200044. 10.1590/1984-3143-ar2020-0044. 33029218 PMC7534573

[cbin70089-bib-0012] Chaipipat, S. , S. Prukudom , K. Sritabtim , et al. 2021. “Primordial Germ Cells Isolated From Individual Embryos of Red Junglefowl and Indigenous Pheasants of Thailand.” Theriogenology 165: 59–68. 10.1016/j.theriogenology.2021.02.010. 33640587

[cbin70089-bib-0013] Chandra, T. , P. A. Ewels , S. Schoenfelder , et al. 2015. “Global Reorganization of the Nuclear Landscape in Senescent Cells.” Cell Reports 10, no. 4: 471–483. 10.1016/j.celrep.2014.12.055. 25640177 PMC4542308

[cbin70089-bib-0014] Chen, J. , Q. Xu , D. Liu , et al. 2023. “CD146 Promotes Malignant Progression of Breast Phyllodes Tumor Through Suppressing DCBLD2 Degradation and Activating the AKT Pathway.” Cancer Communications 43, no. 11: 1244–1266. 10.1002/cac2.12495. 37856423 PMC10631482

[cbin70089-bib-0015] Chen, Q. M. , J. C. Bartholomew , J. Campisi , M. Acosta , J. D. Reagan , and B. N. Ames . 1998. “Molecular Analysis of H2O2‐induced Senescent‐Like Growth Arrest in Normal Human Fibroblasts: p53 and Rb Control G1 Arrest but Not Cell Replication.” Biochemical Journal 332, no. 1: 43–50. 10.1042/bj3320043. 9576849 PMC1219449

[cbin70089-bib-0016] Chen, Y.‐H. , and S. M. Pruett‐Miller . 2018. “Improving Single‐Cell Cloning Workflow for Gene Editing in Human Pluripotent Stem Cells.” Stem Cell Research 31: 186–192. 10.1016/j.scr.2018.08.003. 30099335

[cbin70089-bib-0017] Choi, H. J. , H. C. Lee , and K. S. Kang , et al. 2015. “Production of Interspecific Germline Chimeras via Embryo Replacement1.” Biology of Reproduction 93, no. 2: 36. 10.1095/biolreprod.114.127365. 26063873

[cbin70089-bib-0018] Devenish, C. , A. C. Lees , N. J. Collar , and S. J. Marsden . 2021. “Multi‐Decadal Land Use Impacts Across the Vast Range of An Iconic Threatened Species.” Diversity and Distributions 27, no. 11: 2218–2230. 10.1111/ddi.13395.

[cbin70089-bib-0019] Dicks, N. , V. Bordignon , and G. F. Mastromonaco . 2021. “Induced Pluripotent Stem Cells in Species Conservation: Advantages, Applications, and the Road Ahead.” In iPSCs From Diverse Species, 221–245. Elsevier. 10.1016/B978-0-12-822228-7.00003-5.

[cbin70089-bib-0020] Falagan‐Lotsch, P. , T. S. Lopes , N. Ferreira , et al. 2015. “Performance of PCR‐Based and Bioluminescent Assays for Mycoplasma Detection.” Journal of Microbiological Methods 118: 31–36. 10.1016/j.mimet.2015.08.010. 26296900

[cbin70089-bib-0021] Faria, P. J. , N. M. R. Guedes , C. Yamashita , P. Martuscelli , and C. Y. Miyaki . 2008. “Genetic Variation and Population Structure of the Endangered Hyacinth Macaw (*Anodorhynchus hyacinthinus*): Implications for Conservation.” Biodiversity and Conservation 17, no. 4: 765–779. 10.1007/s10531-007-9312-1.

[cbin70089-bib-0022] Floege, J. , E. Eng , V. Lindner , et al. 1992. “Rat Glomerular Mesangial Cells Synthesize Basic Fibroblast Growth Factor. Release, Upregulated Synthesis, and Mitogenicity in Mesangial Proliferative Glomerulonephritis.” Journal of Clinical Investigation 90, no. 6: 2362–2369. 10.1172/JCI116126. 1361494 PMC443391

[cbin70089-bib-0023] Garvin, A. M. , and L. C. Katwa . 2023. “Primary Cardiac Fibroblast Cell Culture: Methodological Considerations for Physiologically Relevant Conditions.” American Journal of Physiology‐Heart and Circulatory Physiology 325, no. 4: H869–H881. 10.1152/ajpheart.00224.2023. 37624100

[cbin70089-bib-0024] Goetz, R. , K. Dover , F. Laezza , et al. 2009. “Crystal Structure of a Fibroblast Growth Factor Homologous Factor (FHF) Defines a Conserved Surface on FHFs for Binding and Modulation of Voltage‐Gated Sodium Channels.” Journal of Biological Chemistry 284, no. 26: 17883–17896. 10.1074/jbc.M109.001842. 19406745 PMC2719427

[cbin70089-bib-0025] Goetz, R. , and M. Mohammadi . 2013. “Exploring Mechanisms of FGF Signalling Through the Lens of Structural Biology.” Nature Reviews Molecular Cell Biology 14, no. 3: 166–180. 10.1038/nrm3528. 23403721 PMC3695728

[cbin70089-bib-0026] Guedes, N. M. R. 2004. “Araras Azuis: 15 anos de estudos o Pantanal.” In SIMPAN 2004 ‐ Sustentabilidade Regional, edited by B. M. A. Sorian , J. R. B. Sereno , E. L. Sarath , and R. C. M. de Oliveira , 1, 1st ed., 53–61. Embrapa Pantanal.

[cbin70089-bib-1001] Guedes, N. M. R. 2009. "Reproductive Success, Mortality and Growth of Nestlings of the Hyacinth Macaw Anodorhynchus Hyacinthinus (Aves, Psittacidae) in the Pantanal, Brazil. PhD thesis, Universidade Estadual Paulista, Institute of Biosciences.

[cbin70089-bib-0027] Guedes, N. M. R. , and L. H. Harper . 1995. “Hyacinth Macaw in the Pantanal.” In The Large Macaws: Their Care, Breeding and Conservation, edited by J. Abramson , B. L. Speer , and J. B. Thomsen , 1, 1st ed., 394–421. Raintree Publications.

[cbin70089-bib-1002] Guedes, N. M. R. , and G. H. F. Seixas . 2002. “Métodos para estudos de reprodução de Psitacídeos.” In Ecologia e conservação de psitacídeos no Brasil, edited by M. Galetti and M. A. Pizo , 1, 1st ed., 123–139. Melopsittacus Publicações Científicas.

[cbin70089-bib-0028] Guedes, N. M. R. , M. C. B. Toledo , F. M. Fontoura , G. F. da Silva , and R. J. Donatelli . 2022. “Growth Model Analysis of Wild Hyacinth Macaw (*Anodorhynchus hyacinthinus*) Nestlings Based on Long‐Term Monitoring in the Brazilian Pantanal.” Scientific Reports 12, no. 1: 15382. 10.1038/s41598-022-19677-5. 36100629 PMC9470691

[cbin70089-bib-0029] Güney‐Esken, G. , Ö. D. Erol , B. Pervin , et al. 2021. “Development, Characterization, and Hematopoietic Differentiation of Griscelli Syndrome Type 2 Induced Pluripotent Stem Cells.” Stem Cell Research & Therapy 12, no. 1: 287. 10.1186/s13287-021-02364-z. 33985578 PMC8117610

[cbin70089-bib-1003] Ha, S. J. , B. G. Kim , Y. A. Lee , et al. 2016. “Effect of Antioxidants and Apoptosis Inhibitors on Cryopreservation of Murine Germ Cells Enriched for Spermatogonial Stem Cells.” PLoS One 11, no. 8: e0161372. 10.1371/journal.pone.0161372. 27548381 PMC4993461

[cbin70089-bib-0030] Harper, J. M. , M. Wang , A. T. Galecki , J. Ro , J. B. Williams , and R. A. Miller . 2011. “Fibroblasts From Long‐Lived Bird Species Are Resistant to Multiple Forms of Stress.” Journal of Experimental Biology 214, no. 11: 1902–1910. 10.1242/jeb.054643. 21562178 PMC3092728

[cbin70089-bib-0031] Hayashi, M. , V. Zywitza , Y. Naitou , et al. 2022. “Robust Induction of Primordial Germ Cells of White Rhinoceros on the Brink of Extinction.” Science Advances 8, no. 49: eabp9683. 10.1126/sciadv.abp9683. 36490332 PMC9733929

[cbin70089-bib-0032] Hayflick, L. , and P. S. Moorhead . 1961. “The Serial Cultivation of Human Diploid Cell Strains.” Experimental Cell Research 25, no. 3: 585–621. 10.1016/0014-4827(61)90192-6. 13905658

[cbin70089-bib-0033] Hou, X. , T. Zaks , R. Langer , and Y. Dong . 2021. “Lipid Nanoparticles for mRNA Delivery.” Nature Reviews Materials 6, no. 12: 1078–1094. 10.1038/s41578-021-00358-0. 34394960 PMC8353930

[cbin70089-bib-0034] Jimenez, A. G. , and J. B. Williams . 2014. “Cellular Metabolic Rates From Primary Dermal Fibroblast Cells Isolated From Birds of Different Body Masses.” Comparative Biochemistry and Physiology. Part A, Molecular & Integrative Physiology 176: 41–48. 10.1016/j.cbpa.2014.07.009. 25038299

[cbin70089-bib-0035] Jozefczuk, J. , K. Drews , and J. Adjaye . 2012. “Preparation of Mouse Embryonic Fibroblast Cells Suitable for Culturing Human Embryonic and Induced Pluripotent Stem Cells.” Journal of Visualized Experiments 64: e3854. 10.3791/3854-v. PMC3471299 22760161

[cbin70089-bib-0036] Katayama, M. , T. Fukuda , T. Kaneko , et al. 2022. “Induced Pluripotent Stem Cells of Endangered Avian Species.” Communications Biology 5, no. 1: 1049. 10.1038/s42003-022-03964-y. 36280684 PMC9592614

[cbin70089-bib-0037] Katayama, M. , T. Hirayama , T. Tani , K. Nishimori , M. Onuma , and T. Fukuda . 2018. “Chick Derived Induced Pluripotent Stem Cells by the Poly‐Cistronic Transposon With Enhanced Transcriptional Activity.” Journal of Cellular Physiology 233, no. 2: 990–1004. 10.1002/jcp.25947. 28387938

[cbin70089-bib-0038] Katayama, M. , M. Onuma , and T. Fukuda . 2021. “KAv‐1 Is Better Suited to Chick Fibroblast Culture Than DMEM or 199 Media.” The Journal of Poultry Science 58, no. 4: 0200085. 10.2141/jpsa.0200085. PMC8630408 34899023

[cbin70089-bib-1004] Kim, D. H. , J. Lee , Y. Suh , M. Cressman , and K. Lee . 2021. “Research Note: Adipogenic Differentiation of Embryonic Fibroblasts of Chicken, Turkey, Duck, and Quail In Vitro by Medium Containing Chicken Serum Alone.” Poultry Science 100, no. 8: 101277. 10.1016/j.psj.2021.101277. PMC8255238 34198089

[cbin70089-bib-0039] Kim, Y. M. , Y. H. Park , J. M. Lim , H. Jung , and J. Y. Han . 2017. “Technical Note: Induction of Pluripotent Stem Cell‐Like Cells From Chicken Feather Follicle Cells.” Journal of Animal Science 95, no. 8: 3479–3486. 10.2527/jas.2017.1418. 28805906

[cbin70089-bib-0040] Kroglund, I. B. , S. K. K. Eide , J. E. Østnes , R. T. Kroglund , J. E. Frisli , and C. A. Waugh . 2022. “Primary Cell Lines From Feathers and Blood of Free‐Living Tawny Owls (*Strix aluco*): A New In Vitro Tool for Non‐Lethal Toxicological Studies.” Frontiers in Genetics 13: 856766. 10.3389/fgene.2022.856766. 35651947 PMC9149357

[cbin70089-bib-0041] Leão, A. P. A. , A. V. Souza , N. F. Mesquita , L. J. Pereira , and M. G. Zangeronimo . 2021. “Antioxidant Enrichment of Rooster Semen Extenders – A Systematic Review.” Research in Veterinary Science 136: 111–118. 10.1016/j.rvsc.2021.02.005. 33607571

[cbin70089-bib-0042] Lee, S.‐Y. , G.‐W. Huang , J.‐N. Shiung , et al. 2012. “Magnetic Cryopreservation for Dental Pulp Stem Cells.” Cells Tissues Organs 196, no. 1: 23–33. 10.1159/000331247. 22285908

[cbin70089-bib-0043] Di Leonardo, A. , S. P. Linke , K. Clarkin , and G. M. Wahl . 1994. “DNA Damage Triggers a Prolonged p53‐Dependent G1 Arrest and Long‐Term Induction of Cip1 in Normal Human Fibroblasts.” Genes & Development 8, no. 21: 2540–2551. 10.1101/gad.8.21.2540. 7958916

[cbin70089-bib-0044] Lincoln, C. K. , and M. G. Gabridge . 1998. “Cell Culture Contamination: Sources, Consequences, Prevention, and Elimination.” Methods in Cell Biology 57: 49–65. 10.1016/S0091-679X(08)61571-X. 9648099

[cbin70089-bib-0045] Liu, C. X. , R. L. Zhang , J. Gao , et al. 2014. “Derivation of Human Embryonic Stem Cell Lines Without Any Exogenous Growth Factors.” Molecular Reproduction and Development 81, no. 5: 470–479. 10.1002/mrd.22312. 24554631

[cbin70089-bib-0046] Liu, X. , J. Huang , T. Chen , et al. 2008. “Yamanaka Factors Critically Regulate the Developmental Signaling Network in Mouse Embryonic Stem Cells.” Cell Research 18, no. 12: 1177–1189. 10.1038/cr.2008.309. 19030024

[cbin70089-bib-1005] López, M. , R. J. Bollag , J. C. Yu , C. M. Isales , and A. Eroglu . 2016. “Chemically Defined and Xeno‐Free Cryopreservation of Human Adipose‐Derived Stem Cells.” PLoS One 11, no. 3: e0152161. 10.1371/journal.pone.0152161. 27010403 PMC4806986

[cbin70089-bib-0047] Lu, Y. , F. D. West , B. J. Jordan , et al. 2012. “Avian‐Induced Pluripotent Stem Cells Derived Using Human Reprogramming Factors.” Stem Cells and Development 21, no. 3: 394–403. 10.1089/scd.2011.0499. 21970437

[cbin70089-bib-0048] Lu, Y. , F. D. West , B. J. Jordan , R. B. Beckstead , E. T. Jordan , and S. L. Stice . 2015. “Generation of Avian Induced Pluripotent Stem Cells.” Methods in Molecular Biology (Clifton, N.J.) 1330: 89–99. 10.1007/978-1-4939-2848-4_9. 26621592

[cbin70089-bib-0049] lUCN . (2021). IUCN Red List. www.iucnredlist.org.

[cbin70089-bib-0050] Lunardi, V. O. , M. R. Francisco , G. T. Rocha , B. Goldschmidt , and P. M. Galetti Junior . 2003. “Karyotype Description of Two Neotropical Psittacidae Species: The Endangered Hyacinth Macaw, *Anodorhynchus hyacinthinus*, and the Hawk‐Headed Parrot, *Deroptyus accipitrinus* (Psittaciformes: Aves), and Its Significance for Conservation Plans.” Genetics and Molecular Biology 26, no. 3: 283–287. 10.1590/S1415-47572003000300011.

[cbin70089-bib-0052] Martin, G. R. , and H. Rubin . 1974. “Effects of Cell Adhesion to the Substratum on the Growth of Chick Embryo Fibroblasts.” Experimental Cell Research 85, no. 2: 319–333. 10.1016/0014-4827(74)90133-5. 4363950

[cbin70089-bib-0053] Miyaki, C. Y. , N. M. R. Guedes , R. P. Herrera , and A. Wajntal . 1995. “Estudo Da Variabilidade Genética E Da Razão Sexual De Uma Populacão Silvestre De Arara Azul Do Pantanal.” Brazilian Journal of Genetics 18, no. 3: 341.

[cbin70089-bib-0054] Mooney, A. , O. A. Ryder , M. L. Houck , J. Staerk , D. A. Conde , and Y. M. Buckley . 2023. “Maximizing the Potential for Living Cell Banks to Contribute to Global Conservation Priorities.” Zoo Biology 42, no. 6: 697–708. 10.1002/zoo.21787. 37283210

[cbin70089-bib-0055] Moore, D. T. , P. H. Purdy , and H. D. Blackburn . 2006. “A Method for Cryopreserving Chicken Primordial Germ Cells.” Poultry Science 85, no. 10: 1784–1790. 10.1093/ps/85.10.1784. 17012169

[cbin70089-bib-0056] Moralli, D. , M. Yusuf , M. A. Mandegar , S. Khoja , Z. L. Monaco , and E. V. Volpi . 2011. “An Improved Technique for Chromosomal Analysis of Human ES and iPS Cells.” Stem Cell Reviews and Reports 7, no. 2: 471–477. 10.1007/s12015-010-9224-4. 21188651 PMC3073051

[cbin70089-bib-0058] Nogueira, I. P. M. , G. M. J. Costa , and S. M. S. N. Lacerda . 2024. “Avian iPSC Derivation to Recover Threatened Wild Species: A Comprehensive Review in Light of Well‐Established Protocols.” Animals: An Open Access Journal From MDPI 14, no. 2: 220. 10.3390/ani14020220. 38254390 PMC10812705

[cbin70089-bib-0059] Ogata, S. , N. Yorioka , and N. Kohno . 2001. “Glucose and Prednisolone Alter Basic Fibroblast Growth Factor Expression in Peritoneal Mesothelial Cells and Fibroblasts.” Journal of the American Society of Nephrology 12, no. 12: 2787–2796. 10.1681/ASN.V12122787. 11729249

[cbin70089-bib-0060] de Oliveira Furo, I. , R. Kretschmer , P. C. O'Brien , M. A. Ferguson‐Smith , and E. H. C. de Oliveira . 2015. “Chromosomal Diversity and Karyotype Evolution in South American Macaws (Psittaciformes, Psittacidae).” PLoS One 10, no. 6: e0130157. 10.1371/journal.pone.0130157. 26087053 PMC4472783

[cbin70089-bib-0061] Park, S. , D. R. Lee , J. S. Nam , C. W. Ahn , and H. Kim . 2018. “Fetal Bovine Serum‐Free Cryopreservation Methods for Clinical Banking of Human Adipose‐Derived Stem Cells.” Cryobiology 81: 65–73. 10.1016/j.cryobiol.2018.02.008. 29448017

[cbin70089-bib-0062] Park, T. S. , D. K. Jeong , J. N. Kim , et al. 2003. “Improved Germline Transmission in Chicken Chimeras Produced by Transplantation of Gonadal Primordial Germ Cells into Recipient Embryos1.” Biology of Reproduction 68, no. 5: 1657–1662. 10.1095/biolreprod.102.006825. 12606438

[cbin70089-bib-0063] Presti, F. T. , N. M. R. Guedes , P. T. Z. Antas , and C. Y. Miyaki . 2015. “Population Genetic Structure in Hyacinth Macaws (*Anodorhynchus hyacinthinus*) and Identification of the Probable Origin of Confiscated Individuals.” Journal of Heredity 106, no. S1: 491–502. 10.1093/jhered/esv038. 26245784

[cbin70089-bib-0064] Rosselló, R. A. , C.‐C. Chen , R. Dai , J. T. Howard , U. Hochgeschwender , and E. D. Jarvis . 2013. “Mammalian Genes Induce Partially Reprogrammed Pluripotent Stem Cells in Non‐Mammalian Vertebrate and Invertebrate Species.” eLife 2: 00036. 10.7554/eLife.00036. PMC3762186 24015354

[cbin70089-bib-1006] Ryder, O. A. , and M. Onuma . 2018. “Viable Cell Culture Banking for Biodiversity Characterization and Conservation.” Annual Review of Animal Biosciences 6: 83–98. 10.1146/annurev-animal-030117-014556. 29447472

[cbin70089-bib-0066] Sakai, Y. , M. Matsumura , T. Iwao , and T. Matsunaga . 2023. “Culture Methods Focusing on Bile Canalicular Formation Using Primary Human Hepatocytes in a Short Time.” In Vitro Cellular & Developmental Biology. Animal 59, no. 8: 606–614. 10.1007/s11626-023-00805-y. 37682508

[cbin70089-bib-0067] dos Santos Ferreira, B. H. , M. da Rosa Oliveira , J. A. Rodrigues , et al. 2023a. “Wildfires Jeopardise Habitats of Hyacinth Macaw (*Anodorhynchus hyacinthinus*), a Flagship Species for the Conservation of the Brazilian Pantanal.” Wetlands 43, no. 5: 47. 10.1007/s13157-023-01691-6.

[cbin70089-bib-0068] Scalzo, S. , A. K. Santos , H. A. Ferreira , et al. 2022. “Ionizable Lipid Nanoparticle‐Mediated Delivery of Plasmid DNA in Cardiomyocytes.” International Journal of Nanomedicine 17: 2865–2881. 10.2147/IJN.S366962. 35795081 PMC9252585

[cbin70089-bib-0069] Schmittgen, T. D. , and K. J. Livak . 2008. “Analyzing Real‐Time PCR Data by the Comparative CT Method.” Nature Protocols 3, no. 6: 1101–1108. 10.1038/nprot.2008.73. 18546601

[cbin70089-bib-0070] Serrano, M. , A. W. Lin , M. E. McCurrach , D. Beach , and S. W. Lowe . 1997. “Oncogenic ras Provokes Premature Cell Senescence Associated With Accumulation of p53 and p16INK4a.” Cell 88, no. 5: 593–602. 10.1016/S0092-8674(00)81902-9. 9054499

[cbin70089-bib-0071] Stanton, M. M. , E. Tzatzalos , M. Donne , N. Kolundzic , I. Helgason , and D. Ilic . 2019. “Prospects for the Use of Induced Pluripotent Stem Cells in Animal Conservation and Environmental Protection.” Stem Cells Translational Medicine 8, no. 1: 7–13. 10.1002/sctm.18-0047. 30251393 PMC6312526

[cbin70089-bib-0072] Tang, D. , C. Lam , and S. Louie , et al. 2018. “Supplementation of Nucleosides During Selection Can Reduce Sequence Variant Levels in CHO Cells Using GS/MSX Selection System.” Biotechnology Journal 13, no. 1: 1700335. 10.1002/biot.201700335. 28745430

[cbin70089-bib-0073] Tavares, F. S. , C. Martins , F. K. Delella , et al. 2024. “Establishment and Characterization of a Primary Fibroblast Cell Culture From the Amazonian Manatee (*Trichechus Inunguis*).” Animals: An Open Access Journal From MDPI 14, no. 5: 686. 10.3390/ani14050686. 38473072 PMC10931340

[cbin70089-bib-0074] Tonus, C. , K. Cloquette , F. Ectors , et al. 2016. “Long Term‐Cultured and Cryopreserved Primordial Germ Cells From Various Chicken Breeds Retain High Proliferative Potential and Gonadal Colonisation Competency.” Reproduction, Fertility, and Development 28, no. 5: 628. 10.1071/RD14194. 25482458

[cbin70089-bib-0075] Toussaint, O. , E. E. Medrano , and T. von Zglinicki . 2000. “Cellular and Molecular Mechanisms of Stress‐Induced Premature Senescence (SIPS) of Human Diploid Fibroblasts and Melanocytes.” Experimental Gerontology 35, no. 8: 927–945. 10.1016/S0531-5565(00)00180-7. 11121681

[cbin70089-bib-0076] Vilaça, S. T. , J. Dalapicolla , R. Soares , N. M. R. Guedes , C. Y. Miyaki , and A. Aleixo . 2024. “Prioritizing Conservation Areas for the Hyacinth Macaw (*Anodorhynchus hyacinthinus*) in Brazil From Low‐Coverage Genomic Data.” Evolutionary Applications 17, no. 11: e70039. 10.1111/eva.70039. 39564451 PMC11573696

[cbin70089-bib-0077] Woodcock, M. E. , A. A. Gheyas , A. S. Mason , et al. 2019. “Reviving Rare Chicken Breeds Using Genetically Engineered Sterility in Surrogate Host Birds.” Proceedings of the National Academy of Sciences 116, no. 42: 20930–20937. 10.1073/pnas.1906316116. PMC6800374 31575742

[cbin70089-bib-0078] Xi, Y. , Y. Nada , T. Soh , N. Fujihara , and M. Hattori . 2003. “Establishment of Feather Follicle Stem Cells as Potential Vehicles for Delivering Exogenous Genes in Birds.” Journal of Reproduction and Development 49, no. 3: 213–219. 10.1262/jrd.49.213. 14967930

[cbin70089-bib-0079] Yamanaka, S. 2008. “Induction of Pluripotent Stem Cells From Mouse Fibroblasts by Four Transcription Factors.” Cell Proliferation 41, no. Issue S1: 51–56. 10.1111/j.1365-2184.2008.00493.x. 18181945 PMC6496227

[cbin70089-bib-0080] Yang, J. , L. Gao , M. Liu , et al. 2020. “Advanced Biotechnology for Cell Cryopreservation.” Transactions of Tianjin University 26, no. 6: 409–423. 10.1007/s12209-019-00227-6.

[cbin70089-bib-0081] Yao, X. , R. Peng , and J. Ding . 2013. “Cell–Material Interactions Revealed via Material Techniques of Surface Patterning.” Advanced Materials 25, no. 37: 5257–5286. 10.1002/adma.201301762. 24038153

[cbin70089-bib-0082] Yoshioka, N. , and S. F. Dowdy . 2017. “Enhanced Generation of iPSCs From Older Adult Human Cells by a Synthetic Five‐Factor Self‐Replicative RNA.” PLoS One 12, no. 7: e0182018. 10.1371/journal.pone.0182018. 28750082 PMC5531586

[cbin70089-bib-0083] Yu, M. , L. A. Marquez‐Curtis , and J. A. W. Elliott . 2024. “Cryopreservation‐Induced Delayed Injury and Cell‐Type‐Specific Responses During the Cryopreservation of Endothelial Cell Monolayers.” Cryobiology 115: 104857. 10.1016/j.cryobiol.2024.104857. 38350589

[cbin70089-bib-0084] Zalomova, L. V. , and E. E. Fesenko . 2024. “FBS‐Based Cryoprotective Compositions for Effective Cryopreservation of Gut Microbiota and Key Intestinal Microorganisms.” BMC Research Notes 17, no. 1: 168. 10.1186/s13104-024-06836-2. 38898515 PMC11188276

[cbin70089-bib-0085] Zhai, W. , D. Yong , J. J. El‐jawhari , et al. 2019. “Identification of Senescent Cells in Multipotent Mesenchymal Stromal Cell Cultures: Current Methods and Future Directions.” Cytotherapy 21, no. 8: 803–819. 10.1016/j.jcyt.2019.05.001. 31138507

[cbin70089-bib-0086] Zhang, T. , H. Yin , Y. Li , et al. 2024. “Optimized Lipid Nanoparticles (LNPs) for Organ‐Selective Nucleic Acids Delivery In Vivo.” IScience 27, no. 6: 109804. 10.1016/j.isci.2024.109804. 38770138 PMC11103379

[cbin70089-bib-0087] Zhao, J. , H. N. Hao , R. L. Thomas , and W. D. Lyman . 2001. “An Efficient Method for the Cryopreservation of Fetal Human Liver Hematopoeitic Progenitor Cells.” Stem Cells 19, no. 3: 212–218. 10.1634/stemcells.19-3-212. 11359946

[cbin70089-bib-0088] Zywitza, V. , E. Rusha , D. Shaposhnikov , et al. 2022. “Naïve‐Like Pluripotency to Pave the Way for Saving the Northern White Rhinoceros From Extinction.” Scientific Reports 12, no. 1: 3100. 10.1038/s41598-022-07059-w. 35260583 PMC8904600

