# Supplementary figures and images for: Establishment, Characterization, and Cryopreservation of Feather Follicle Fibroblast Lines From Hyacinth Macaw (Anodorhynchus hyacinthinus)
Source: Cell Biol Int. 2025 Oct 2;49(12):1730–50. doi: 10.1002/cbin.70089 (PMC12605831; doi:10.1002/cbin.70089)

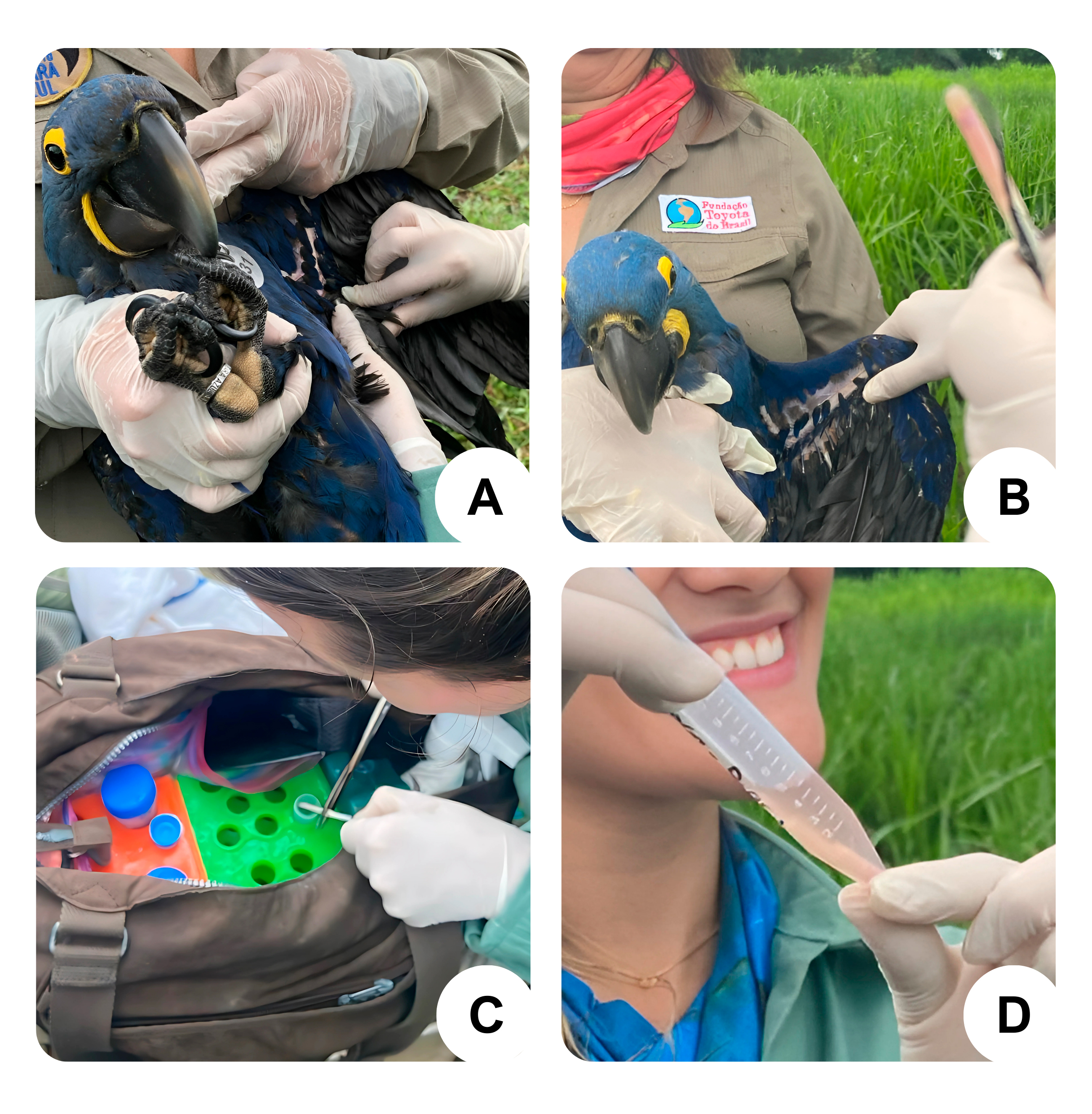

Supplement: Supplementary file 1 — Supplementary Figure 1. Sequential steps for in‐field collection of Hyacinth Macaw feathers from nestlings. The procedure begins with the selection of an optimal feather (A), followed by careful extraction of the calamus (B). The calamus is then separated from the feather and placed in a centrifuge tube for washing (C). Finally, the feather samples are immersed in culture medium for transport under controlled conditions (D). [file CBIN-49-1730-s002.tif]

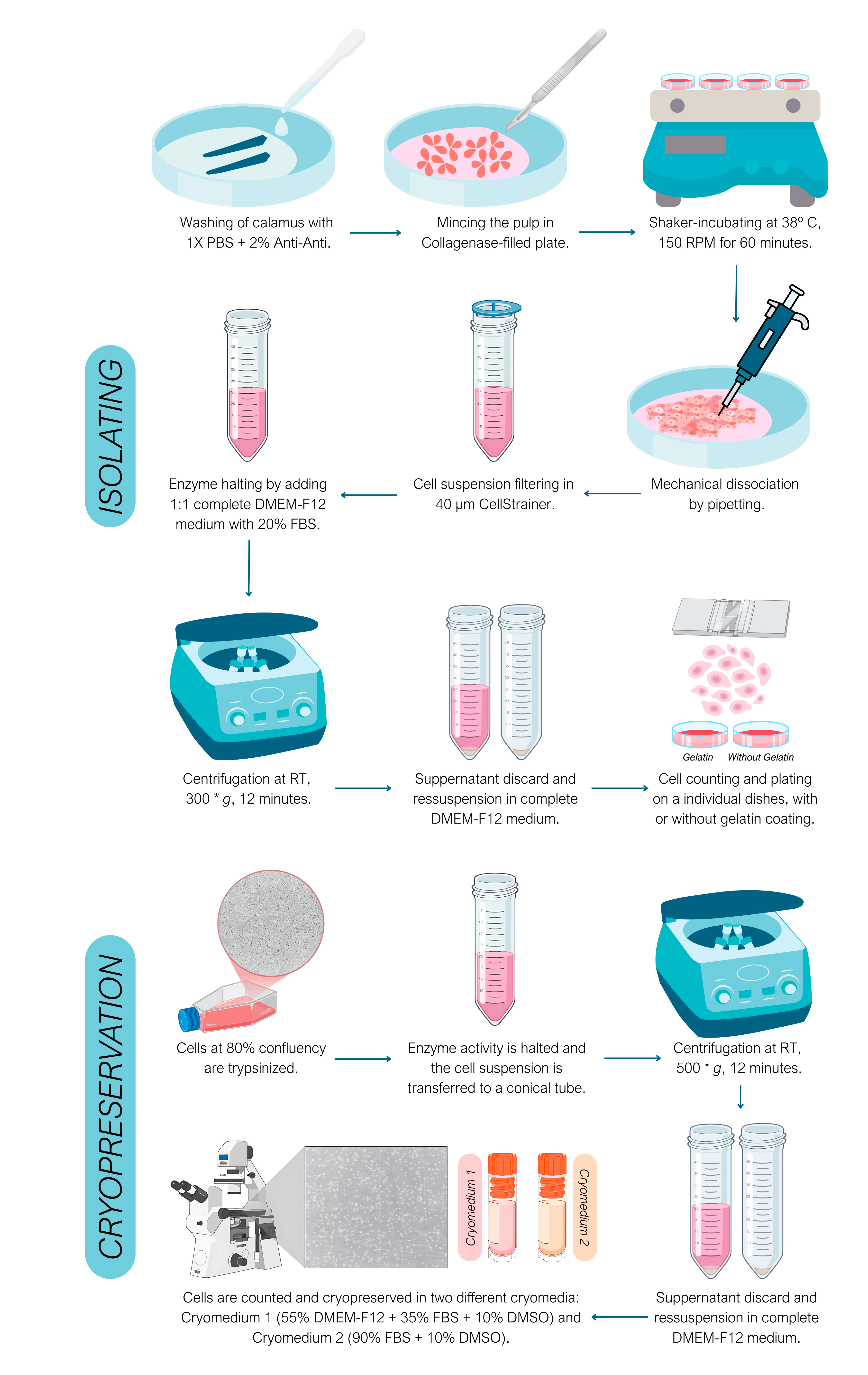

Supplement: Supplementary file 2 — Supplementary Figure 2. Overview of A. hyacinthinus feather follicle fibroblasts (FFFs) isolation and cryopreservation workflow. [file CBIN-49-1730-s001.tif]
